# The Novel lncRNA ENST00000530525 Affects ANO1, Contributing to Blood–Brain Barrier Injury in Cultured hCMEC/D3 Cells Under OGD/R Conditions

**DOI:** 10.3389/fgene.2022.873230

**Published:** 2022-06-08

**Authors:** Wen Jiang, Jie Li, Yuefang Cai, Wenchen Liu, Mei Chen, Xiaoying Xu, Minzhen Deng, Jingbo Sun, Lihua Zhou, Yan Huang, Shuang Wu, Xiao Cheng

**Affiliations:** ^1^ Department of Second Institute of Clinical Medicine, Guangzhou University of Traditional Chinese Medicine, Guangzhou, China; ^2^ Department of Anesthesiology, Guangdong Provincial Hospital of Traditional Chinese Medicine/The Second Affiliated Hospital of Guangzhou University of Chinese Medicine, Guangzhou, China; ^3^ Department of Neurology, Guangdong Provincial Hospital of Traditional Chinese Medicine/The Second Affiliated Hospital of Guangzhou University of Chinese Medicine, Guangzhou, China; ^4^ Department of Anatomy, Sun Yat-Sen School of Medicine, Sun Yat-Sen University, Shenzhen, China; ^5^ State Key Laboratory of Dampness Syndrome of Chinese Medicine, The Second Affiliated Hospital of Guangzhou University of Chinese Medicine, Guangzhou, China; ^6^ Guangdong Provincial Key Laboratory of Research on Emergency in TCM, Guangzhou, China

**Keywords:** ischemic stroke, ANO1, lncRNA, ENST00000530525, oxygen-glucose deprivation and reperfusion, human cerebrovascular endothelial cells

## Abstract

Ischemic stroke (IS) is a major neurological disease with high fatality and residual disability burdens. Long noncoding RNAs (lncRNAs) have been found to play an important role in IS. However, the roles and significance of most lncRNAs in IS are still unknown. This study was performed to identify differentially expressed (DE) lncRNAs using a lncRNA microarray in whole blood samples of patients suffering from acute cerebral ischemia. Bioinformatics analyses, including GO, KEGG pathway enrichment analysis, and proximity to putative stroke risk location analysis were performed. The novel lncRNA, ENST00000530525, significantly decreased after IS. Furthermore, we evaluated lncRNA ENST00000530525 expression in cultured hCMEC/D3 cells under oxygen-glucose deprivation/reoxygenation (OGD/R) conditions using fluorescent *in situ* hybridization (FISH) and quantitative real-time polymerase chain reaction (RT–qPCR) analysis. To investigate the function of lncRNA ENST00000530525, its over-expression (OE) and negative control (NC) plasmids were transfected into hCMEC/D3 cells, and cell viability was detected by a cell counting kit-8 (CCK-8) assay after OGD/R. LncRNA ENST00000530525 and ANO1 expression were investigated using RT–qPCR and immunofluorescence. For blood–brain barrier (BBB) permeability, FITC-dextran transendothelial permeability assay and tight junction (TJ) protein immunofluorescence assays were performed. There were 3352 DE lncRNAs in the blood samples of acute IS patients. The validation results were consistent with the gene chip data. The GO and KEGG results showed that these lncRNAs were mainly related to oxygen and glucose metabolism, leukocyte transendothelial migration, mitophagy and cellular senescence. Among these, lncRNA ENST00000530525 was the most highly downregulated lncRNA and it was mapped within the IS-associated gene anoctamin-1 (ANO1). We further found that lncRNA ENST00000530525 was downregulated in hCMEC/D3 cells under 4 h OGD and 20 h reoxygenation (OGD4/R20) conditions. Upregulating lncRNA ENST00000530525 by plasmid transfection decreased cell viability while increasing ANO1 expression and it contributed to BBB injury in hCMEC/D3 cells after OGD4/R20. The lncRNA ENST00000530525 might play deleterious roles in post-stroke pathogenesis. These results show that some DE lncRNAs in humans participate through characteristic roles in post-stroke pathogenesis; thus, the roles and significance of some novel lncRNAs in IS warrant further study.

## Introduction

Stroke is a high risk factor for death and disability worldwide. IS is the most prevalent, accounting for up to 80% of all stroke cases (Wang et al., 2017). Hypertension, diabetes, dyslipidemia, atrial fibrillation, and obesity are prevalent among Chinese individuals aged 40 years and older (Li et al., 2017). This makes stroke a very important health issue.

Cerebral blood flow interruption initiates a cascade of complex and poorly understood pathophysiologic processes, including oxidative stress, inflammation and apoptosis (Schaukowitch and Kim, 2014; Ren and Yang, 2018). Therefore, many scientists have turned to epigenetics to understand the molecular mechanisms, regulation of the complex stroke-induced pathophysiological processes as well as the elucidation of new therapeutic targets and the design of new treatment modalities (Dharap et al., 2012; Liu et al., 2018)). The most promising theme thus far is the alteration of the expression of some noncoding RNAs that could participate in post-stroke processes, both neuroprotective and neurodegenerative.

Long noncoding RNAs (lncRNAs) are more than 200 nucleotides long and do not encode any proteins. Previous studies showed lncRNAs could modulate cell survival, inflammation, and angiogenesis (Bao et al., 2018). They appear to perform these varied functions by interacting with multiple elements involved in the central dogma of biology, such as other RNAs, DNA, and proteins (Akella et al., 2019). Recently, a number of abnormally expressed lncRNAs have been identified in IS though microarrays, high-throughput deep sequencing, and RNA-seq (Bao et al., 2018). However, the importance and significance of most lncRNAs remain poorly understood and warrant further study in IS.

Therefore, this research was performed to evaluate lncRNA expression in patients’ whole blood samples after acute cerebral ischemia. Bioinformatics analyses were also used for the dysregulated genes to reveal the predicted connections and functions of the DE lncRNAs. Furthermore, we found that lncRNA ENST00000530525 is highly decreased in stroke patients relative to controls, and the high conservation of lncRNA ENST00000530525 suggests that it may be important in stroke pathology.

LncRNA ENST00000530525 is located intragenically with its neighbor gene ANO1. However, the role of lncRNA ENST00000530525 and the regulatory mechanism linking lncRNAs and ANO1 signaling in stroke remain enigmatic. ANO1 (also known as transmembrane protein 16A, TMEM16A) widely expresses in eukaryotes and is a molecular indicator of calcium-activated chloride channels. Recent studies have shown that the upregulation of ANO1 is related to the development of many diseases, such as IS (Wang et al., 2012; Wu et al., 2014; Deng et al., 2016). Recently, it was found that ANO1 is strongly expressed in cerebrovascular endothelial cells, IS-induced BBB injury is accompanied by upregulation of ANO1. Inhibiting ANO1 significantly rescued OGD/R-induced downregulation of TJ proteins and upregulation of BBB permeability *in vitro* (Liu et al., 2019). And the present studies showed that ANO1 affects the functions of brain capillary endothelial cells, which are involved in BBB functions (Suzuki et al., 2020; Liu et al., 2021; Ma et al., 2021; Suzuki et al., 2021).

As we know, BBB is part of the neurovascular unit that plays a vital role in regulating blood-to-brain flux of endogenous and exogenous xenobiotics and associated metabolites. Because of the TJ protein between the endothelial cells of the BBB, blood-borne substances and cells have restricted access to the brain. Under IS condition, the BBB can be destroyed and loss of the TJ integrity, followed by the extravasation of blood components into the brain and compromise of normal neuronal function, future research on the BBB is likely to reveal promising potential therapeutic targets for protecting the BBB and improving patient outcome after IS (Jiang et al., 2018).

Based on the above, the underlying mechanisms and targets of lncRNA ENST00000530525 in hCMEC/D3 cells under OGD/R injury were investigated, which may provide a novel idea for us to better understand the function and significance of lncRNAs in the pathophysiological mechanism of IS. Further studies of these lncRNAs in biological systems after IS may provide opportunities to identify biomarkers and new therapeutic targets.

## Materials and Methods

### Study Subjects

Ten patients with a diagnosis of IS who were seen at Guangdong Province Traditional Chinese Medical Hospital from September 2016 to November 2017 along with 20 control subjects were recruited to join the study. The protocol was approved by the ethics committee of Guangdong Provincial Hospital of Chinese Medicine (B2016-149-01). After explaining study details, patients and their immediate caregivers or significant others provided written informed consent to participate. There was no incentive to participate.

Patients’ histories, examinations, and evaluations were collected after qualified neurologists diagnosed them as acute IS. Standard stroke assessments performed at our hospital included computed tomographic (CT), electrocardiography (ECG), magnetic resonance imaging (MRI), carotid Doppler, Holter monitor and computed tomographic angiography or magnetic resonance arteriogram of extracranial and intracranial vessels according to the clients’ clinical data. Acute IS patients who presented with a rare pathogenesis other than atherosclerosis and cardiogenic stroke, psychopathology, pregnancy and lactation or those patients who were in other clinical trials when they suffered a stroke were not eligible to participate in this study. The age and sex of the 20 control subjects were matched to those of the stroke patients; the control patients were without a history of stroke and did not have severe heart disease, cardiac insufficiency, hepatitis, renal insufficiency, respiratory failure, malignant tumor or gastrointestinal bleeding.

### Derivation and Validation Group

Ten IS patients and 20 non-stroke subjects were recruited to detect DE lncRNAs and randomly distributed them to the derivation group or the validation group. All patients’ race, sex, age, and vascular risk factors (diabetes mellitus, hypertension, and hyperlipidemia) were matching. Exclusion criteria included prior stroke; treatment with thrombolysis or anticoagulant before sample collection; hemorrhagic infarction; infection before or after stroke; subarachnoid hemorrhage; recreational drug abuse; dialysis; cancer; and immunosuppressive therapy for blood abnormalities or steroids. The validation set was used to prove the microarray analysis results. The primer sequences used are provided in Supplementary Table S1.

### Blood Collection and RNA Isolation

Venipuncture was performed once on all study participants, and their whole blood was drawn into EDTA tubes (Jingxin Biotechnology Co., Ltd., China). For stroke patients, blood collection time varied from a few hours to 2 weeks after symptom onset. For the control samples, the time of extraction was not applicable. And then the tubes was rapidly frozen with liquid nitrogen and put at -80°C until use. RNA isolation was performed by using TRIzol (Invitrogen, United States), which was added to every cryopreserved serum supernatant sample according to the protocol.

### Array Hybridization

Arraystar Human lncRNA V4.0 chip (Arraystar, United States) was used, which can test 20,730 protein-coding and 40,173 lncRNA transcripts. Sample marking and chip hybridization were based on the recommended experimental procedure of Agilent One-Color Microarray-Based Gene Expression Analysis (Agilent Technology, United States). Briefly, a mRNA-ONLY™ Eukaryotic mRNA Isolation Kit (Epicenter, United States) was used to remove the rRNA from mRNA, which was then amplified and transcribed into cRNA with fluorescence by random priming. The marked cRNAs was purified by the RNeasy Mini Kit (Qiagen 74104, Germany). The RNA was quantified by a NanoDrop ND-1000 spectrophotometer (NanoDrop Technologies, Thermo Fisher, United States), and an Agilent 2100 Bioanalyzer (Agilent, United States) was used to assess the quality of the samples. The GenePix 4000B chip scanner (Agilent, United States) was used to wash and process arrays. DAT file formats were used to save raw values. The scanned results were transformed into digital data and then saved.

### Differential Expression Analysis

To identify DE lncRNAs, cuffdiff was used for the differential expression analysis. Only probes with a fold-change higher than 2 and *p* < 0.05 were selected as significantly DE lncRNAs or mRNAs. To obtain the overall characteristics of the lncRNA and mRNA expression profiles, we used the R package for hierarchical clustering analysis of normalized values of all DE lncRNAs to generate heatmaps. Bioinformatics analyses, including the Gene Ontology project (GO, http://geneontology.org/), the database Kyoto Encyclopedia of Genes and Genomes (KEGG) pathway enrichment analysis (http://www.kegg.jp/kegg/), and network analysis, were also performed for the identified DE genes. Through a literature review, NONCODE, UCSC Genome Browser, and OMIM^®^—Online Mendelian Inheritance in Man^®^ databases, we further explained the biology of the differential lncRNAs.

### Data Normalization

The original data were exported by GeneSpring GX v12.1 software (Agilent, United States). The data were normalized by preprocess Core in the R package, and log2 transformation was performed to obtain the final normalized data. The standardized data were further analyzed by screening high-quality probes marked as detected in at least three samples out of six. Hierarchical clustering was conducted using R script. Finally, the lncRNAs expressed at significantly different levels between the two groups of samples were screened by volcano plots.

### hCMEC/D3 Cell Culture

HCMEC/D3 cells (FuHeng Biology, FH1110, Shanghai, China) were cultured in endothelial cell medium (ECM,ScienCell, Carlsbad, United States) and added fetal bovine serum (FBS, Gibco, Australia) into ECM to making its concentration to 10%, and then incubated the cells in humidified atmosphere at 37°C with 5% CO_2_. All experiments with hCMEC/D3 cells were used below 15 passages.

### OGD/R Condition

To mimic acute IS *in vitro*, hCMEC/D3 cells were subjected to OGD/R. The establishment of OGD model in this paper refers to the literatures (Fan et al., 2021; Xu et al., 2021). When the cells became confluent, glucose-free DMEM (Cienry, Zhejiang, China) was used to replace the culture medium, and the cells were put into an anaerobic incubator (94% N_2_, 5% CO_2_, 1% O_2_) for 2, 4, 6, and 8 h. After incubation, the cells were returned to regular conditions and normal medium for reoxygenation. The total time of oxygen glucose deprivation and reoxygenation was 24 h.

### CCK-8 Assay

The cell counting kit-8 assay (Dojindo, Tokyo, Honshu, Japan) was used to detect cell viability. All of the procedures followed the manufacturer’s instructions. Cell suspensions (100 μl, 1 × 10^4^ cells/mL) were added to a 96-well plate. After incubating with NC or OE (MOI = 50 nM) in 96-well microtiter plates, CCK-8 solution (10 μl) was put into 96-well plate after OGD for 4 h and reoxygenation for 20 h. Finally, absorbance at 450 nm of the wells was measured by the microplate reader (SYNERGY H1, BioTek), and five replicate wells were assessed in each experiment.

### RNA FISH

Subcellular localization of the lncRNA ENST00000530525 was detected by the FISH Kit (RiboBio, China). The Cy3-labeled lncRNA ENST00000530525 probe and the control probe 18S were all designed by RiboBio (Guangzhou, China). Briefly, 4% paraformaldehyde was used to fix hCMEC/D3 cells for 15 min at room temperature and washed with PBS three times for 5 min each time. The cells were incubated in proteinase K for 5 min at 4°C and washed as before. Then, prehybridization buffer was added to the cells for incubation at 37°C and then they were incubated in hybridization buffer with specific probes for lncRNA ENST00000530525 and 18S at 37°C overnight. After extensive washing with SSC, the cell nucleus was stained by DAPI for 5 min. The images were took by the fluorescence microscope (Nikon Ti2-E, Japan).

### Plasmid Transfection

RiboBio company (Guangzhou, China) provided the lncRNA ENST00000530525 (OE) overexpression plasmid, negative control plasmid (NC) and transfection kit (riboFECT™ CP, C10511-05). HCMEC/D3 cells were cultured and incubated for 3 days with OE or NC (MOI = 50 nM), which was then replaced by glucose-free DMEM for OGD 4 h. RT–qPCR was used to detect the transfection efficiency of lncRNA ENST00000530525.

### FITC-Dextran Transendothelial Permeability Assay

In brief, cell suspension was added into polycarbonate 12-well Transwell inserts with a 0.4 μm pore size (Corning, United States) at a density of 4 × 10^4^ cells/cm^2^. When cells grown to approximately 70% confluence, they were transfected with plasmid. Then FITC-labeled dextran (0.1 mg/ml, MW, 70000, Sigma-Aldrich) was added to the upper chamber and 500 μL PBS was added to the lower cavity after OGD/R. Then we put them in a cell incubator for 5 min. The microplate reader was used to analyze the supernatant from the lower chamber (excitation at 490 nm, emission at 520 nm).

### Quantitative Real-Time PCR for lncRNA and mRNA

The protocol of RT-qPCR are as follows. We used TRIzol (Thermo Fisher Scientific, Waltham, MA, United States) to separate total RNA from hCMEC/D3 cells. For reverse transcriptase, we used cDNA synthesis kits (Takara, Dalian, China) to synthesize cDNA. SYBR^®^Premix Ex Taq™II (Takara, Dalian, China) was used for amplification. The internal reference is human β-actin gene. Comparative quantification was performed by using the 2^−ΔΔCt^ method. The primer sequences used are shown in Supplementary Table S1.

### Immunofluorescence

Cells were cultured on 24-well plates for immunofluorescence staining at a seeding density of 7 × 10^4^ cells per well (Shin et al., 2016). For immunofluorescent staining, 4% paraformaldehyde was used to fix the cell at 4°C overnight. After blocking with 5% BSA, the first antibodies: rabbit monoclonal anti-ANO1 (1:200), rabbit monoclonal anti-ZO1 (1:200), rabbit anti-occludin (1:100), and rabbit anti-claudin-5 (1:200) were added to the cells at 4°C overnight. Then the cells were incubated with the corresponding secondary antibody in a dark chamber at 37°C for 1 h. The antibodies and secondary antibodies were obtained from Abcam. After stained with DAPI (1:500, Beyotime), the fluorescence microscope (Nikon TI2-E) was used to take the images.

### Transfection and Luciferase Assays

293T cells were inoculated in 24-well plates and cultured until the degree of cell fusion reached over 60%. Plasmid transfections for luciferase assays were performed with 1 µg plasmid and 2 µL X-tremegene HP reagent (ROCHE, Switzerland) as described by the manufacturer. The expression of fluorescent-labeled genes on the plasmid was observed 24–48 h after transfection to determine the transfection efficiency. And then 48 h post transfection, luminescence was detected using the Dual-Luciferase Reporter Assay System as described by the manufacturer (Promega, United States). Data were normalized to the Renilla luminescence and presented relative to control transfected group.

### Statistical Analysis

All quantitative experiments were performed at least three times, and the data are showed as the mean ± SD. Statistical tests were performed using SPSS 24.0 statistical software and Graphed Prism 6 was used for graphic representations. The comparisons of patient characteristics between two groups were analyzed by the chi-square test (sex, hypertension history, diabetes history, smoking history, cardiovascular history, and hyperlipidemia) and unpaired two-tailed Student’s t-test (age). One-way ANOVA was used to analyze the comparisons among multiple groups. *p* < 0.05 was considered statistically significant.

## Results

### Characteristics

The characteristics of 10 stroke patients and 20 non-stroke cases are shown in Table 1. There were significantly more stroke patients with a positive history of hypertension, diabetes, and smoking.

**TABLE 1:** Demographics and the distribution of the comorbid conditions in the study sample.

Characteristics	Non-stroke group	Stroke group
	(*n* = 20)	(*n* = 10)
Age (years)	64.54 ± 11.83	63.58 ± 10.20
Gender (Male)	14 (70%)	8 (80%)
Hypertension history	10 (50%)	7 (70%)*
Diabetes history	1 (5%)	3 (30%)*
Smoking history	3 (15%)	4 (40%)*
Cardiovascular disease history	3 (15%)	1 (5%)
Hyperlipidemia	5 (25%)	5 (5%)

The table shows the age of study participants along with the distribution of their comorbid conditions such as hypertension, diabetes, smoking, cardiovascular disease and hyperlipidemia.

### Differential lncRNA Expression and GO and KEGG Analyses Among IS Patients versus Non-stroke Patients

**FIGURE 1:**
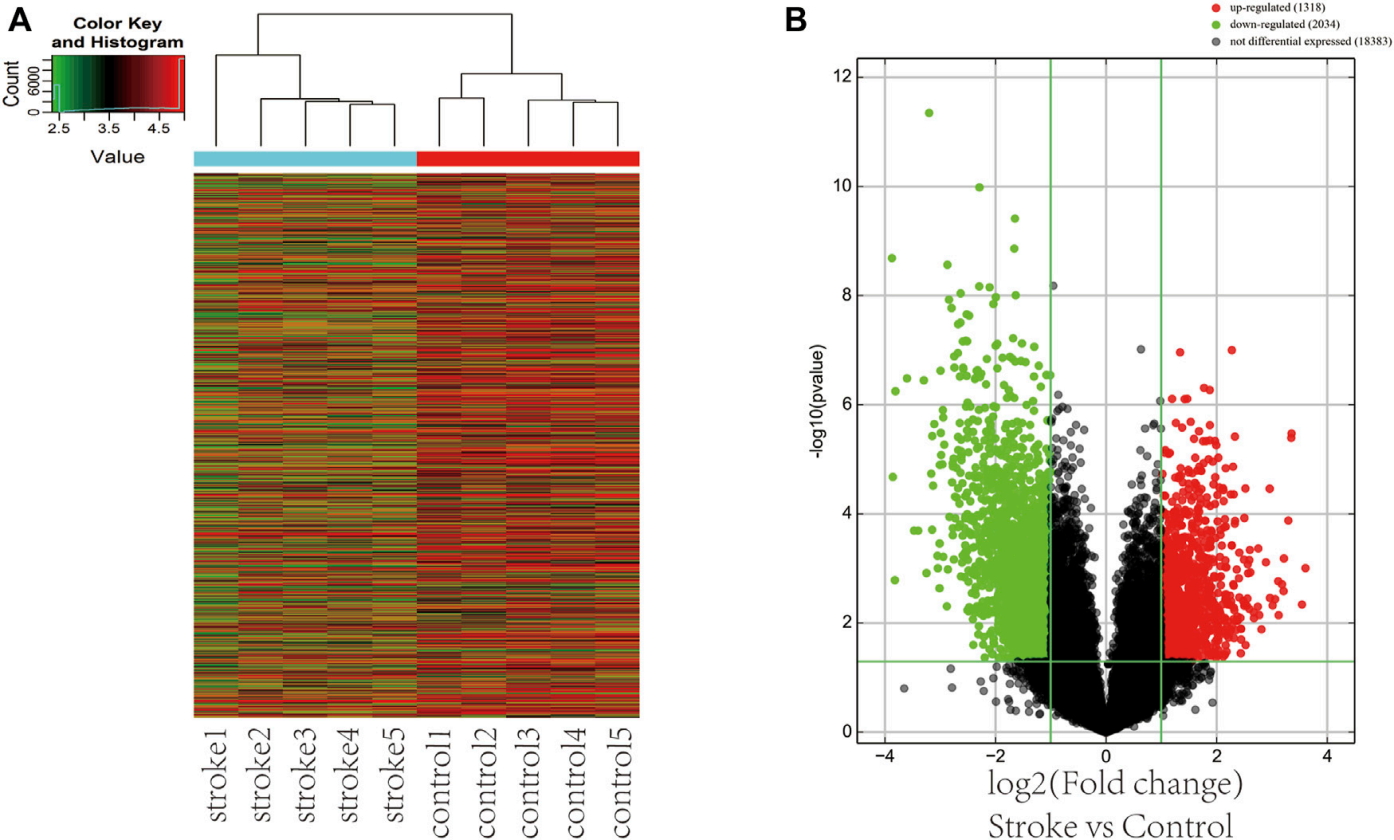
The DE lncRNAs among ischemic stroke patients versus non-stroke controls. **(A)** Hierarchical clustering shows different lncRNA expressions between stroke and nonstroke patients. **(B)** Volcano plot of the DE lncRNAs in stroke patients and nonstroke controls. Red points in the plot are statistically significant differentially upregulated lncRNAs, while green represents downregulated lncRNAs.

We used the Arraystar Human lncRNA V4.0 chip to analyze the DE lncRNAs between two groups. Unsupervised hierarchical clustering analysis generated from the expression profile of the lncRNAs showed the different expression levels between non-stroke and stroke patients (Figure 1A). Volcano plots were used to show the statistically significant differences expression of lncRNAs. There were 3352 DE lncRNAs, with 1318 upregulated and 2034 downregulated lncRNAs (Figure 1B). The top 10 most significantly up and downregulated lncRNAs are listed in Table 2. T120068 (fold change 12.2021) was the most highly upregulated lncRNAs. ENST00000530525 (fold change -14.63) was the most highly downregulated lncRNAs.

**TABLE 2:** The top 10 highly up- and downregulated lncRNAs.

Seq-Name	*p*-value	Fold change	Regulation	Gene symbol
A				
T120068	0.0010	12.2021	up	G028344
T346481	0.0045	11.6843	up	G081575
uc002vbb.3	0.0001	10.2441	up	BC047484
T333126	0.0001	10.2030	up	G078089
ENST00000530249	0.0001	9.8429	up	CTD-3138F19.1
ENST00000597530	0.0007	9.3130	up	RP11-251M1.1
T224664	0.0026	9.2702	up	G051903
ENST00000447488	0.0019	9.0912	up	AC073254.1
NR_046,612	0.0071	8.7159	up	HTR2A-AS1
T344288	0.0017	8.6688	up	G080966
B				
ENST00000530525	0.0001	14.6300	down	RP11-805J14.3
T198099	0.0001	14.4622	down	G045690
ENST00000523658	0.0016	14.0950	down	RP11-402L5.1
T235708	0.0001	12.1118	down	G054213
T328914	0.0002	11.0822	down	G077138
NR_132,119	0.0002	10.5270	down	GTF2IP20
T024196	0.0001	9.8059	down	G005224
ENST00000458252	0.0012	9.4884	down	AC123886.2
T056136	0.0001	9.1733	down	G012959
TCONS_00010056	0.0001	8.9389	down	XLOC_004,504

**FIGURE 2:**
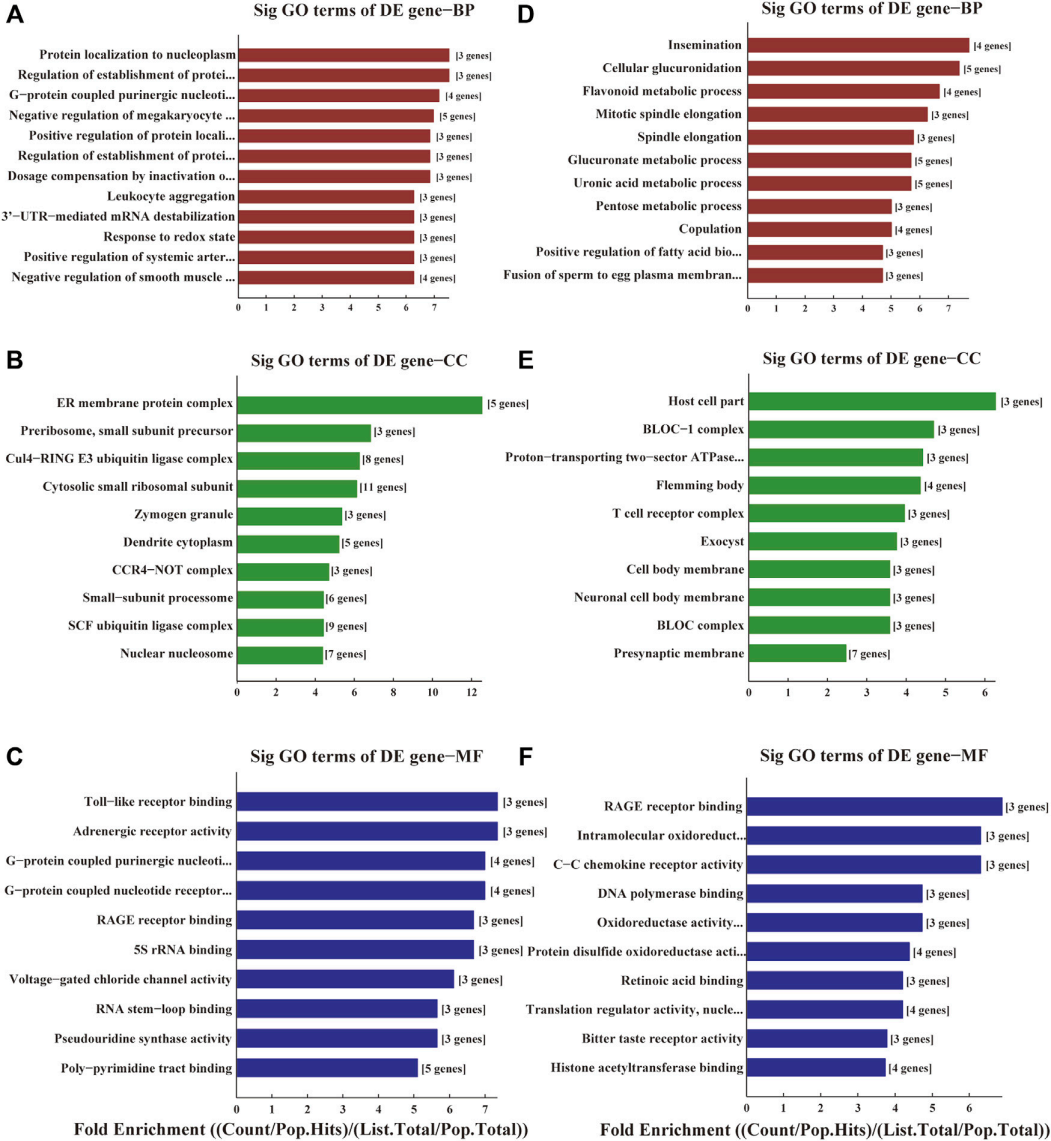
GO analyses of the DE genes among ischemic stroke patients versus non-stroke controls. GO terms of Biological Process (BP), Cellular Component (CC) and Molecular Function (MF) for the DE upregulated **(A–C)** and downregulated genes **(D–F).**

The functions of these lncRNAs were predicted with the co-expressed mRNAs using GO and KEGG pathway annotations. In GO annotation, the upregulated genes were mainly related to ER membrane protein complexes, Toll-like receptor binding, protein localization to the nucleoplasm (Figures 2A–C). The downregulated genes were manly related to RAGE receptor binding, intramolecular oxidoreductase activity, cellular glucuronidation, cellular glucuronation and T cell receptor complexes (Figures 2D–F).

**FIGURE 3:**
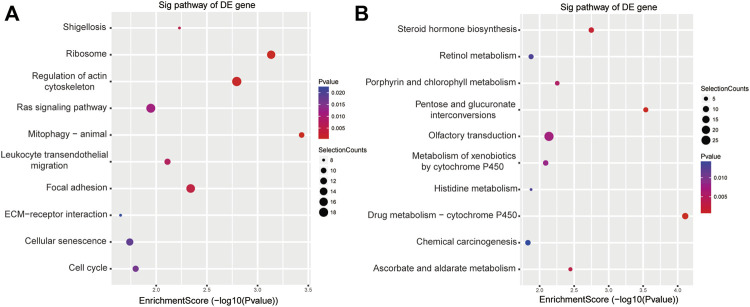
KEGG analyses of the DE genes among ischemic stroke patients versus non-stroke controls. KEGG terms for the DE upregulated **(A)** and downregulated genes **(B)**.

The results of KEGG enrichment showed that these DE genes were related to pathways in pathophysiological processes underpinning acute cerebral infarction, such as pentose and glucuronate interconversions, regulation of the actin cytoskeleton, RAS signal pathway, focal adhesion, leukocyte transendothelial migration, mitophagy and cellular senescence (Figures 3A,B).

### Validation of the DE lncRNAs

**FIGURE 4:**
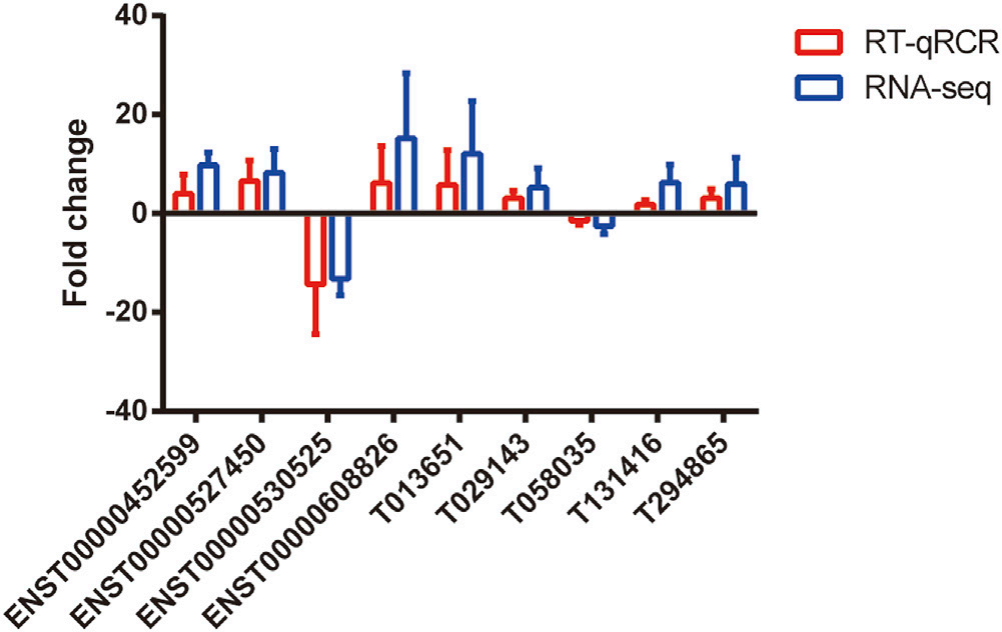
Validation of differential expression of lncRNAs. RT-qPCR was used to verify the expression levels of these selected lncRNAs (*p* < 0.05). The blue points represent RT-qPCR, while the red points represent the gene chip of RNA-seq. *n* = 5.

We selected nine DE lncRNAs at random and used RT–qPCR analysis to prove our gene chip performance (Figure 4). There were seven upregulated (ENST00000452599, ENST00000527450, ENST00000608826, T013651, T029143, T131416, and T294865) and two downregulated lncRNA transcripts (ENST00000530525 and T058035). The RT–qPCR results were consistent with the gene chip data in the two groups.

### Biological Interpretation-Proximity to Putative Stroke Genes

Among the upregulated lncRNAs, ASHGV40004037 was annotated to NR_046612 that mapped to the putative ischemic risk locus HTR2A at cytoband 13q14.2. Similarly, ASHGV40013775 was annotated to NR_002312, which has genomic coordinates 45 kb downstream of the PARP2 cerebral ischemic injury-associated gene at 14q11.2. ASHGV40056557 is expressed as NR_037932, which maps to the stroke-associated genes apolipoprotein C and E within 420 kb, while T111838 (ASHGV40056142) maps at 14q32.33 within metastasis-associated protein 1 (MTA1), a transcriptional regulator-associated gene modifing master chromatin. Additionally, T294865 (ASHGV40042838) mapped within 350 kb of another gene ADAMTS2 associating with stroke at 5q35.3, while T297706 (ASHGV40043041) mapped to Sirtuin 5 (SIRT5) within 6 kb at 6p23. Among the downregulated lncRNAs, the annotation of ASHGV40008778 identified the validated lncRNA ENST00000530525, which is within anoctamin-1 (ANO1), a chloride ion channel gene related to cerebrovascular remodeling. ENST00000395996 (ASHGV40001758) is within VPS13B and is associated with cerebral ischemic injury. Furthermore, NR_045414 (ASHGV40037155) at 4p16.3 mapped within 8 kb of HTT and is associated with post-stroke depression (PSD), while ENST00000416061 (ASHGV40053990)’s genomic coordinates were 3.4 kb downstream of SUV39H1, a histone methyltransferase related to stroke (Table 3).

**TABLE 3:** Up and downregulated lncRNAs and proximity to putative stroke genes.

Probe name	lncRNA ID	Cyto-band	Gene in region	lncRNA Up-/down-stream	lncRNA Distance (kb) from gene	FC
ASHGV40004037	NR_046612	13q14.2	HTR2A	within	within	8.7159
ASHGV40013775	NR_002312	14q11.2	PARP2	down	45	6.1135
ASHGV40056557	NR_037932	19q13.32	APOC2	down	416.9	4.7449
			APOC4	down	419.2	
			APOE	down	461.9	
ASHGV40056142	T111838	14q32.33	MTA1	within	within	4.6273
ASHGV40042838	T294865	5q35.3	ADAMTS2	up	346.4	4.0617
ASHGV40043041	T297706	6p23	SIRT5	down	5.7	3.9144
ASHGV40008778	ENST00000530525	11q13.3	ANO1	within	within	−14.6300
ASHGV40001758	ENST00000395996	8q22.2	VPS13B	within	within	−14.0950
ASHGV40037155	NR_045414	4p16.3	HTT	down	7.6	−6.3636
ASHGV40053990	ENST00000416061	XP11.23	SUV39H1	down	3.4	−4.9941

Significant DE lncRNA probe sets in stroke vs. non-stroke groups and their proximity to putative stroke genes (“-” means downregulated, fold change (FC) ≥ 2.0, *p* < 0.05).

### LncRNA ENST00000530525 was Located Intragenically with ANO1

To futher detect the expression trend of lncRNA ENST00000530525 in stroke patients, we expanded the samples and randomly chose 40 blood samples from stroke patients and 20 blood samples from controls. LncRNA ENST00000530525 expression was significantly decreased in IS group compared with the control group (Supplementary Figure S1). To further research the function of lncRNA ENST00000530525 in stroke pathology, the noncoding nature of lncRNA ENST00000530525 was first confirmed by coding-potential analysis (Supplementary Figure S2) (Qu et al., 2016). Then, we checked Ensembl (Abdillahi et al., 2012), an online database used to predict gene location (Yates et al., 2020), and found that the lncRNA ENST00000530525 is located intragenically with ANO1 (Supplementary Figure S3A). ANO1 is strongly expressed in cerebrovascular endothelial cells (Liu et al., 2019), and study showed that ANO1 contributed to the proliferation and migration of brain capillary endothelial cells, which are involved in BBB functions (Suzuki et al., 2020). We also checked STRING (Lo et al., 2007), an online database, to summarize the network of predicted associations for this protein (Szklarczyk et al., 2015). ANO1 is known to have strong links to epithelial ion channels, such as cystic fibrosis transmembrane conductance regulator (CFTR) and the Bestrophin family (Supplementary Figure S3B). Thus, we further speculated that lncRNA ENST00000530525 and ANO1 are involved in the pathology of stroke. Because ANO1 is strongly expressed in cerebrovascular endothelial cells, in the following study, the cells undergo OGD/R was chosen to mimic IS.

### LncRNA ENST00000530525 was DownRegulated in hCMEC/D3 Cells After OGD/R

**FIGURE 5:**
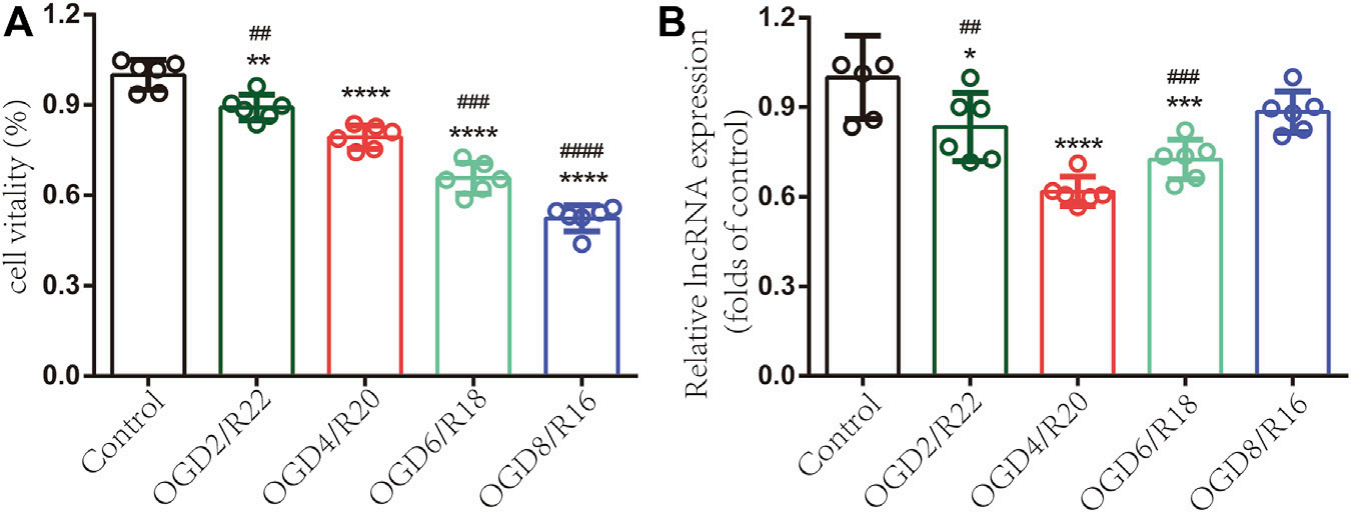
LncRNA ENST00000530525 is downregulated in hCMEC/D3 cells under OGD/R condition. Cells were exposed to OGD for 2, 4, 6 or 8 h and reoxygenate for 22, 20, 18 or 16 h. Each bar represents a different OGD duration and reoxygenation time (e.g., OGD2/R22 represents 2 h of OGD and 22 h of reoxygenation). **(A)** The cell vitality worsened as the OGD time increased **(B)** LncRNAs highly downregulated under OGD4/R20 conditions. *p < 0.05, **p < 0.01, ****p < 0.0001 (vs. control); ##*p* < 0.01, ###*p* < 0.001, ####*p* < 0.0001 (vs. OGD4/R20), *n* = 6, one-way ANOVA.

To investigate lncRNA ENST00000530525 expression in hCMEC/D3 cells after OGD/R injury, CCK-8 assays were used to test cell vitality. The results indicated that the longer the OGD time was, the worse the cell vitality (Figure 5A). The RT–qPCR results showed lncRNA ENST00000530525 expression was significantly downregulated in hCMEC/D3 cells after OGD/R compared with control cells, which is consistent with the results of ChIP sequencing (Figure 5B). The expression of lncRNA ENST00000530525 was highly downregulated after 4 h OGD and 20 h reoxygenation.

### Upregulating lncRNA ENST00000530525 Expression Affected the Viability of hCMEC/D3 Cells After OGD/R

**FIGURE 6:**
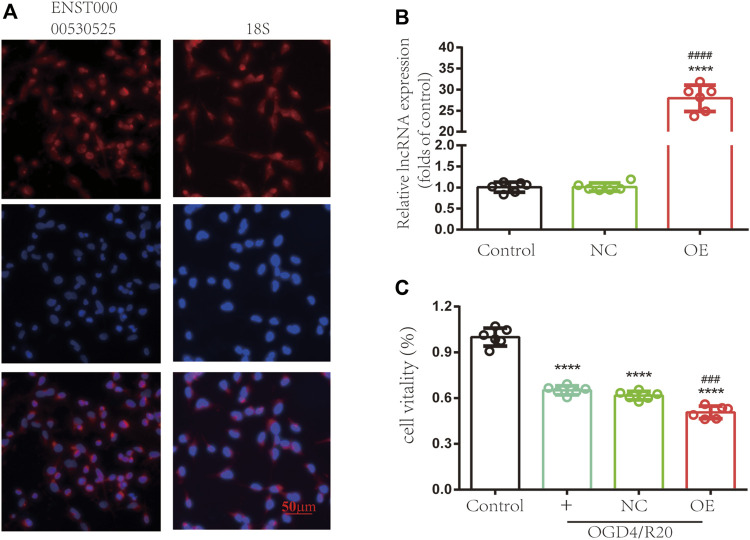
Upregulating the expression of lncRNA ENST00000530525 affects the vitality of hCMEC/D3 cells. **(A)** FISH showed that ENST00000530525 was mainly distributed in the cytoplasm, similar to the control group 18S. Scale bar: 50 μm. **(B)** The transduction efficiency detected by RT–qPCR. **(C)** The CCK-8 assay revealed cell vitality. NC: negative control; OE: over expression; OGD4/R20: 4 h of OGD and 20 h of reoxygenation. ****p < 0.0001 (vs. control); ###*p* < 0.001, ####*p* < 0.0001 (vs. NC), *n* = 6, one-way ANOVA.

To explore the functions of lncRNA ENST00000530525 in hCMEC/D3 cells after OGD/R, lncRNA ENST00000530525 was upregulated by transfection of an overexpression plasmid. The location of lncRNA ENST00000530525 was tested by FISH. The results showed lncRNA ENST00000530525 was mostly distributed in the cytoplasm, similar to the control group-18S, which means overexpression plasmid transfection would effectively promote the lncRNA’s function (Figure 6A).

The RT–qPCR results uncovered lncRNA ENST00000530525 expression in the OE group was effectively upregulated by 28-fold compared to it in the NC group after 72 h of transfection in hCMEC/D3 cells, while there was no difference between the control and NC-treated groups (Figure 6B). This result suggested that the transient plasmid transfection of lncRNA into cultured hCMEC/D3 cells could selectively upregulate lncRNA ENST00000530525 expression.

We investigated whether overexpression of lncRNA ENST00000530525 can influence the viability of hCMEC/D3 cells after OGD/R. The results revealed the cell viability was 1.00 ± 0.05 in untreated cells as a control, 0.65 ± 0.03 in the normal control group, 0.62 ± 0.03 in the NC-treated group, and 0.51 ± 0.04 in the OE-treated group. Statistical analysis showed the cell viability of the OE-treated group was decreased compared to that of the NC-treated group after OGD/R injury (Figure 6C), which indicated that overexpression of lncRNA ENST00000530525 in cultured hCMEC/D3 cells could lead to more severe OGD/R ischemia injury.

### Upregulating lncRNA ENST00000530525 Increased the Expression of ANO1

**FIGURE 7:**
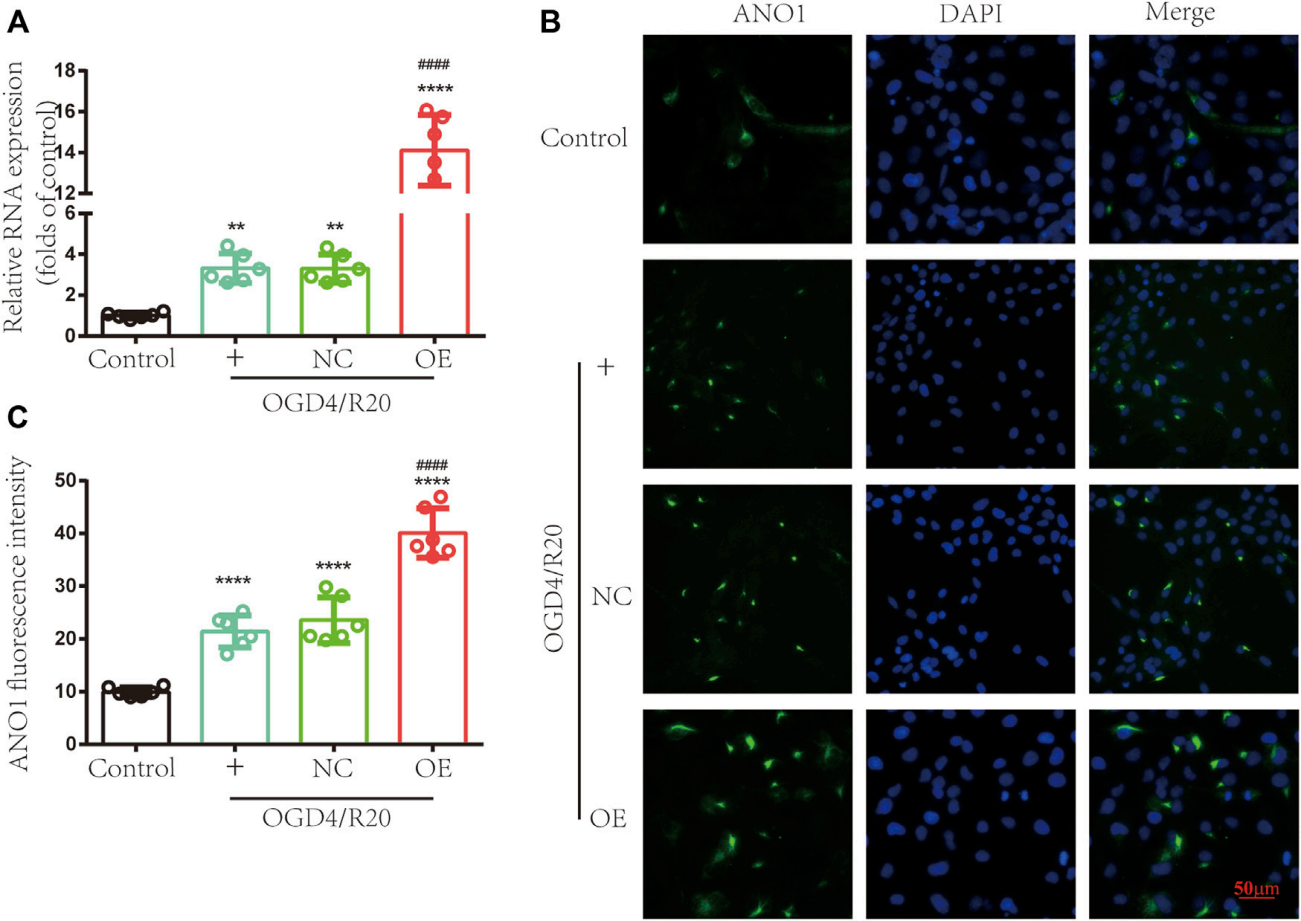
Upregulating lncRNA ENST00000530525 increased the expression of ANO1. The effects of lncRNA ENST00000530525 upregulation by plasmid on the gene and protein expression of ANO1 in hCMEC/D3 cells after OGD4/R20 conditions. **(A)** The RT–qPCR results show that ANO1 increased after OGD4/R20, which increased more significantly when lncRNA ENST00000530525 was upregulated by the plasmid. **(B,C)** The immunofluorescence results show that transfecting a plasmid into hCMEC/D3 cells resulted in an increase in ANO1 protein expression. Scale bar: 50 μm. NC: negative control; OE: over expression; OGD4/R20: 4 h of OGD and 20 h of reoxygenation. **p < 0.01, ****p < 0.0001 (vs. Control); ####*p* < 0.0001 (vs. NC), *n* = 6, one-way ANOVA.

To test whether upregulation of lncRNA ENST00000530525 affects the expression of ANO1, RT–qPCR and immunofluorescence were used. RT–qPCR results showed the expression of ANO1 in the OGD4/R20 group was upregulated. Meanwhile, the ANO1 expression in the OE group was effectively upregulated by 4.27-fold compared to that in the NC-treated cells after 72 h of transfection in hCMEC/D3 cells, but there was no significant difference between OGD4/R20 group and NC group (Figure 7A).

Immunofluorescence showed that the protein expression of ANO1 increased after OGD4/R20. Meanwhile, when lncRNA ENST00000530525 was upregulated by transfection of the overexpression plasmid, ANO1 protein expression in the OE group increased almost 2-fold compared to that in the NC group, while there was no significant difference between OGD4/R20 group and NC group (Figures 7B,C). These results demonstrated that altered expression of lncRNA ENST00000530525 could influence the expression of ANO1 in hCMEC/D3 cells after OGD4/R20.

### Targeted Increases in lncRNA ENST00000530525 Deteriorated BBB Disruption After OGD4/R20

**FIGURE 8:**
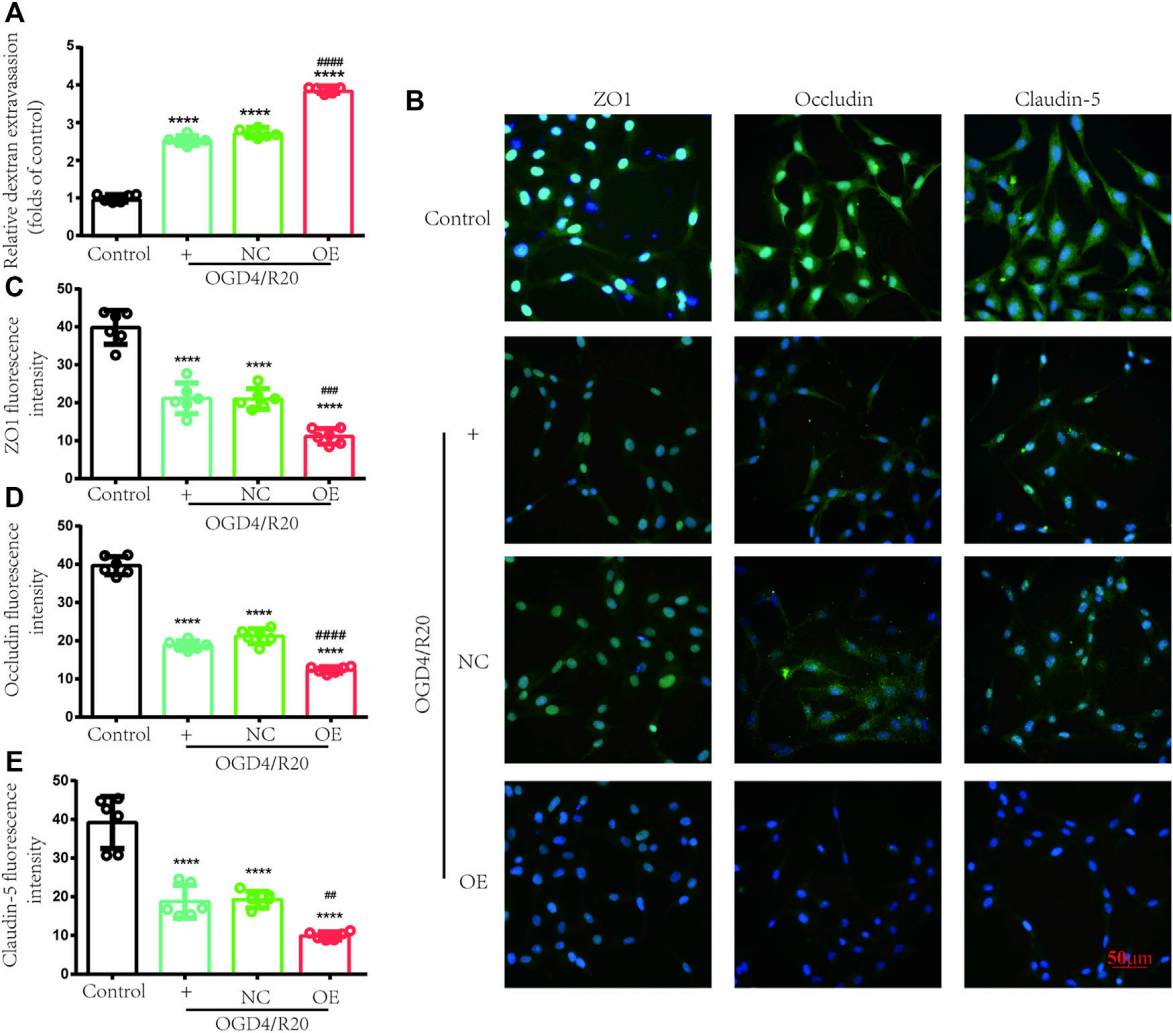
Increasing lncRNA ENST00000530525 deteriorated transendothelial permeability under OGD/R condition. **(A)** FITC-dextran leakage was used to quantificat transendothelial permeability. **(B–E)** Representative immunofluorescence indensity of ZO-1, occludin and claudin-5. Scale bar: 50 μm. NC: negative control; OE: over expression; OGD4/R20: 4 h of OGD and 20 h of reoxygenation. ****p < 0.0001 (vs. Control); #*p* < 0.001, ##*p* < 0.01; ###*p* < 0.001, ####*p* < 0.0001 (vs. NC), *n* = 6, one-way ANOVA.

FITC-dextran permeability was used to evaluate BBB permeability. The results showed in the OGD4/R20 group FITC-dextran permeability was upregulated compared to the control group. However, FITC-dextran permeability in the OE group was effectively upregulated by 1.41-fold compared to that in the NC-treated cells 72 h after plasmid transfection, while there was no difference between the OGD4/R20 group and NC group (Figure 8A).

Endothelial cells, which were connected by TJ proteins, are a major component of BBB. TJ proteins (ZO-1, occludin, and claudin-5) were evaluated by immunofluorescence to assess the state of BBB. After OGD4/R20, the fluorescence intensity of these proteins significantly decreased compared to the control group. Meanwhile, lncRNA ENST00000530525 overexpression futher decreased the fluorescence intensity of these proteins compared to the NC group (Figures 8B–E). These results also showed that lncRNA ENST00000530525 overexpression downregulated the expression of ZO-1, occludin and claudin-5 under OGD4/R20 condition.

### LncRNA ENST00000530525 Not Directly Bind to 3′UTR of ANO1

In order to investigate whether lncRNA ENST00000530525 directly bind to 3′UTR of ANO1 and affect its expression, a double luciferase assay was used. Luc-ANO1-NC, Luc-ANO1-mimic, lncRNA-NC, and lncRNA-OE plasmids were constructed and transfected into 293T cells. After 48 h transfection, firefly luminescence and renilla luminescence were detected. However, there was no significant difference between Luc-ANO1-mimic group and Luc-ANO1-NC group when lncRNA ENST00000530525 was over-expressed (*p* < 0.05 but the difference was less than 20%), which indicated that lncRNA could not directly bind to the target gene ANO1 and affect its expression (Supplementary Figure S4).

## Discussion

**FIGURE 9:**
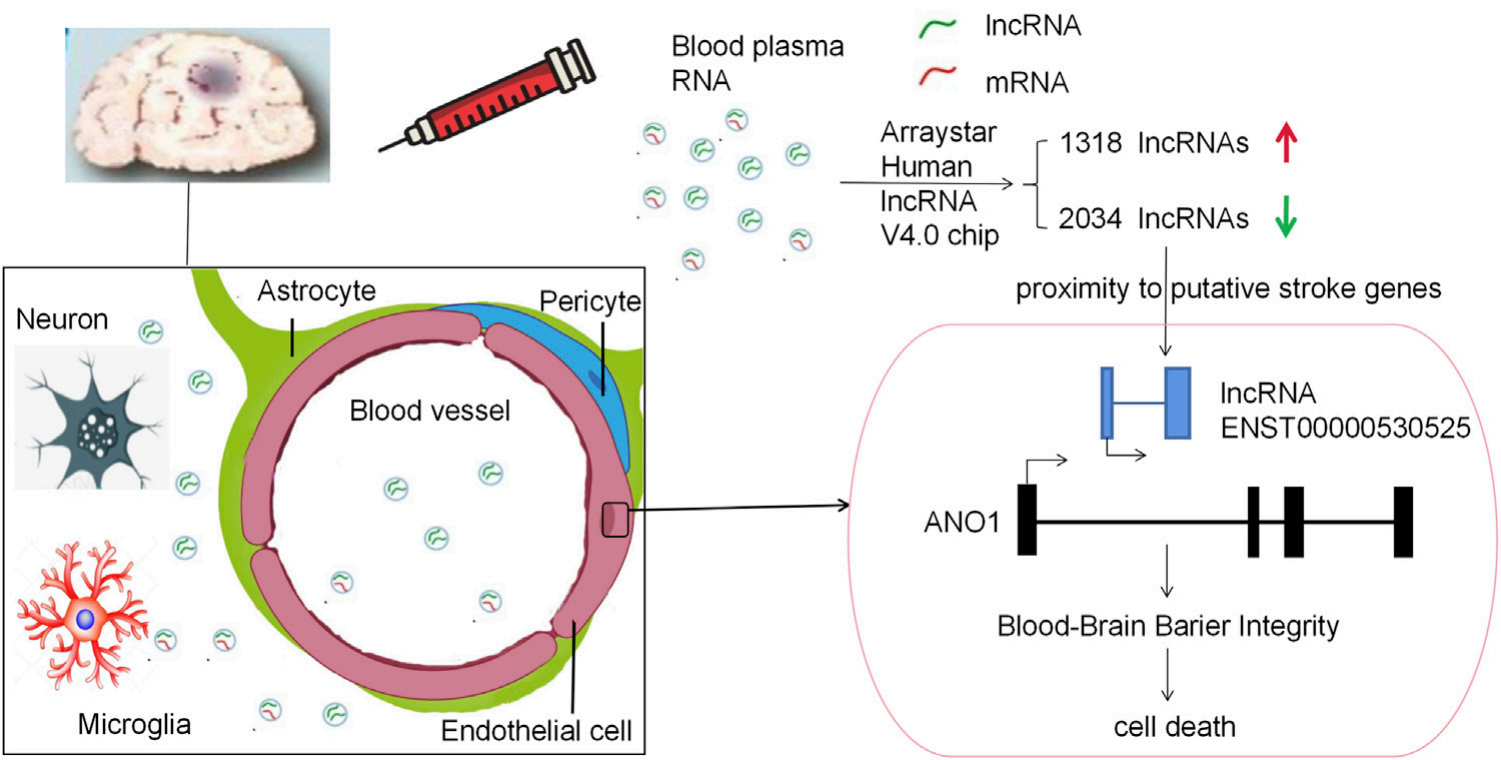
Schematic diagram of this paper. This study used a RNA-chip sequencing in whole blood samples of patients suffering from acute cerebral ischemia to identify DE lncRNAs. The Arraystar Human lncRNA V4.0 chip results showed there were 3352 lncRNA transcripts DE, with 1318 upregulated and 2034 downregulated lncRNAs. LncRNA ENST00000530525 was the highest downregulated lncRNA, while ANO1 was in proximity to the putative stroke gene. Upregulating lncRNA ENST00000530525 improved the expression of ANO1, which further destroyed the BBB integrity, in the hCMEC/D3 cells after OGD4/R20.

In our study, we uncovered lncRNA ENST00000530525 was downregulated after OGD4/R20, but ANO1 was upregulated. After IS, ANO1 was showed to be upregulated and to destroy the BBB integrity by regulating the NF-kB signaling pathway (Liu et al., 2019). This study was consistent with these researches. Notably, when lncRNA ENST00000530525 was upregulated by plasmid transfection, the RNA and protein expression of ANO1 was also increased, while the vitality of hCMEC/D3 cells and the BBB integrity were inhibited. This indicated that lncRNA ENST00000530525 might affect its neighbor gene ANO1 to play an important role in BBB integrity (Figure 9).

Stroke is a high risk factor for death and disability worldwide. However, the narrow time window for treatment of its standard therapy and the difficulty in making a quick diagnosis in most low-resource medical facilities (no brain imaging) indicates a need to understand the molecular regulation of stroke and the identification of its biomarkers. Such knowledge would enable the identification of biomarkers among the DE proteins and mRNAs as well as to provide new opinions to the mechanisms of stroke pathogenesis in patients (ENCODE Project Consortium, 2012; Falcione et al., 2020). Consequently, this study identified several stroke-induced changes in the expression of some circulating lncRNAs and their probable target genes.

Among the upregulated lncRNAs, NR_002312 at 14q11.2 is 45 kb away from poly (ADP-ribose) polymerase-2 (PARP2). PARP2 is responsive to DNA damage and drives cell death pathways in IS (Kofler et al., 2006). Additionally, lncRNA NR_037,932 (upregulated) was mapped to APOC2 and APOC4 within 420 kb and APOE within 462 kb at 19q13.32. APOE is central to the transport and metabolism of lipids and plays an important role in both cerebral IS and coronary heart disease (Satizabal et al., 2018). APOC4 correlates with stroke recovery, while APOC2 is involved in the pathophysiology of post-stroke depression (Zhan et al., 2014; Plubell et al., 2020). These associations may be key to the functions of lncRNAs in the evolution of stroke and can serve as potential biomarkers if pursued further. Another significant DE lncRNA, T297706, is within 6 kb of SIRT5 at 6p23, which increases blood–brain barrier permeability by degrading occludins (Diaz-Cañestro et al., 2018) and it promotes arterial thrombosis *via* endothelial PAI-1 expression (Liberale et al., 2020).

Among the downregulated lncRNAs, ENST00000395996 is within vacuolar protein sorting 13 homolog B (VPS13B). VPS13B participates in the pathoevolution of atherosclerosis-induced IS (Ruan et al., 2020). The HTT gene is less than 8 kb away from the downregulated lncRNA NR_045414, while the upregulated lncRNA is within the HTR2A gene. Both HTT and HTR2A are associated with post-stroke disease (Kim et al., 2012). The highly downregulated lncRNA ENST00000530525 is within the ANO1 gene. It is plausible that the lncRNA ENST00000530525 may affect the expression of the ANO1 gene after IS due to proximity. Our guess was identified by plasmid transfection assays, which mean upregulation of the lncRNA ENST00000530525 could influence the expression of ANO1. But the double luciferase assay result showed, the lncRNA ENST00000530525 could not directly bind to and regulate its neighbor gene ANO1.

Previous studies in rodents revealed the mechanisms of action of some of the dysregulated lncRNAs. There are four archetypes of molecular functions that lncRNAs execute: decoys, guides, signals, and scaffolds (Wang and Chang, 2011). For instance, *cis*-regulation, which means lncRNA can somehow bind to adjacent DNA in chromosomes and regulate its expression, is one kind of signal and plays a crucial role in stroke (Yan et al., 2017). LncRNA Peril acts in this manner to positively regulate the expression of two genes 1.5 million base pairs (Mb) away from its transcription location (Groff et al., 2018). Accordingly, it is possible that other cis-lncRNAs may regulate cerebral ischemic stroke genes in this way, as several significantly DE lncRNAs in our study mapped within or to the neighboring genes associated with IS (Table 3).

Recent studies have provided intriguing evidence for decoy lncRNAs. LncRNA U90926 binds directly to malate dehydrogenase 2 (MDH2), competitively inhibiting the binding of MDH2 to the untranslated region (UTR) of CXCL2, thereby protecting the mRNA of CXCL2 from MDH2-mediated attenuation (Chen et al., 2021). As for signal, the lncRNAs in this archetype can act as markers of functionally significant biological events. For example, HOTTIP, a lncRNA found at the distal end of the human HOXA cluster, directly binds the adaptor protein WDR5 and targets WDR5/MLL complexes across HOXA, driving histone H3 lysine 4 trimethylation and gene transcription (Wang et al., 2011). Another archetype of lncRNA is the guide—RNA binds proteins (usually transcription factors), then directs the protein complex to specific targets. Engreitz found that 5 of 12 lncRNA loci regulate their neighboring gene transcription. In most of these lncRNA, local effects are mediated by enhancer-like functions of DNA elements, while in one locus perhaps by recruit more transcription-associated factors (Engreitz et al., 2016). In conclusion, the lncRNA ENST00000530525 may recruit transcription factors or enhancers to promote ANO1 gene transcription. In this study, the mechanism by which lncRNA ENST00000530525 affects its target gene ANO1 requires further research.

There are still some problems being worthy of consideration and in-depth exploration in this study. The basic disease of stroke patients is complex and diverse in clinical, the incidence of ischemia stroke is common in the middle-aged and elderly who have high-risk factors (such as Hypertension, diabetes, and hyperlipidemia). The race, sex, age, and vascular risk factors (diabetes mellitus, hypertension, and hyperlipidemia) were matching of all patients enrolled in this study and there were significantly more stroke patients with a positive history of hypertension, diabetes, and smoking in Table 1, the expression of the novel lncRNA ENST00000530525 in stroke patients without risk factors has not been verified due to the limitation of sample, However, we are randomly sampling 40 blood samples from stroke patients and 20 blood samples from controls to detect the expression trend of lncRNA ENST00000530525, which is downregulated in stroke group compared with the control group (See Supplementary Figure S1). Moreover, the gene chip results were gotten from patients’ blood, but our further validation used hCMEC/D3 cells. It is not clear how the reduced circulating levels of lncRNA in patients’ blood is linked to the downregulation of its expression in the hCMEC/D3 cells, and the relationship between them needs to be further studied. In addition, there are varying times of BBB permeability detection according to the literatures. In Cowan’s study, FITC-dextran was added to the inserts, which were then transferred every 5 min over 30 min to a series of collecting wells to measure the BBB permeability (Cowan and Easton, 2010). Gerhartl used FITC-dextran to assess BBB permeability after transferring 30 min (Gerhartl et al., 2020). While in Liu’s study, the BBB permeability test is performed for 20 min (Liu et al., 2019). There were still other researchers added FITC-Dextran the upper chamber and then incubated for 1 h to test (Ni et al., 2017; Bergman et al., 2021). BBB permeability test just performed for 5 min in our study, the test time may need further verification. In this study, if the expression of ANO1 and cell viability under upregulating and downregulating lncRNA conditions by transfection, and also ANO1 expression in lncRNA overexpressed cells under normal conditions (not just OGD/R pathological condition) were investigated, the conclusion would be more convincing.

Due to the relatively recent discoveries, the expression and functions of the majority of lncRNAs in the post-stroke brain are largely unknown. However, numerous lncRNAs are emerging as important regulators of transcription and translation. These findings of dysregulated lncRNAs may help to guide evidence-based preventive measures and the search for a cure. Further studies will be needed to establish whether these remaining lncRNAs can modulate theis neighboring and stroke-associated genes *in vivo* or *in vitro*.

## Data Availability

The datasets presented in this study can be found in online repositories. The names of the repository/repositories and accession number(s) can be found below: GEO—GSE198710

## Abbreviations

ANO1, Anoctamin-1; BBB, Blood-brain barrier; CCK-8, cell counting kit-8; CT, Computed tomographic; CFTR, Cystic fibrosis transmembrane conductance regulator; CSF, Codon substitution frequency; DE, Differential expressed; ECG, Electrocardiograph; FE, Feature Extraction; FISH, Fluorescent *in situ* hybridization; IS, Ischemic stroke; lncRNAs, Long noncoding RNAs; NC, Negative control; OE, Over-expression; ORF, Open reading frame; OGD/R, Oxygen-glucose deprivation/reoxygenation; RT-qPCR, Quantitative real-time polymerase chain reaction; TMEM16A, Transmembrane protein 16A; TJ, Tight junction.
