# Supplementary material for: The Novel lncRNA ENST00000530525 Affects ANO1, Contributing to Blood–Brain Barrier Injury in Cultured hCMEC/D3 Cells Under OGD/R Conditions
Source: Front Genet. 2022 Jun 8;13:873230. doi: 10.3389/fgene.2022.873230 (PMC9213740; doi:10.3389/fgene.2022.873230)

raw picture

Fig.7B   ANO1

Control group

1 2 3 4 5 6

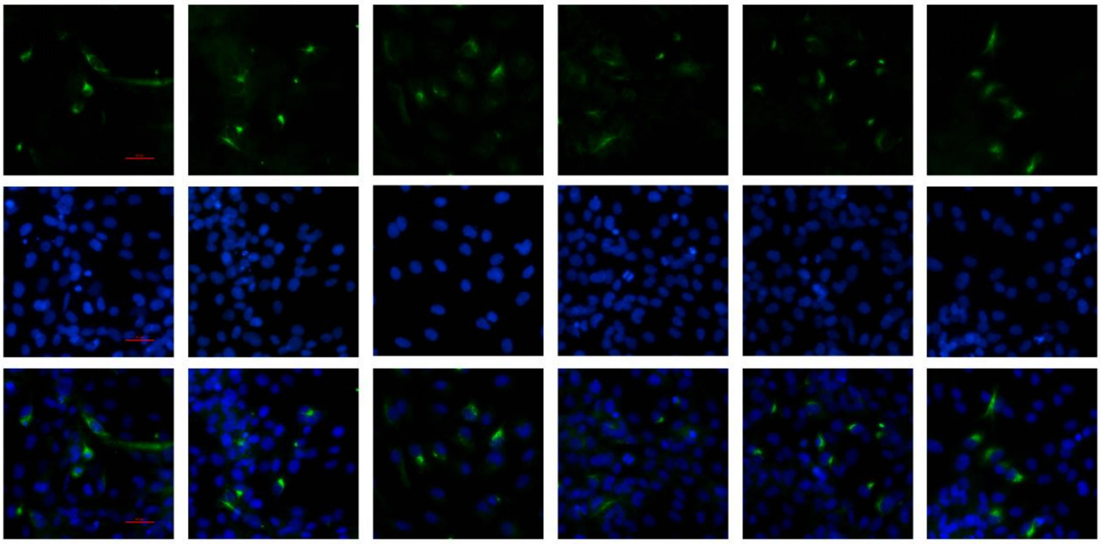

OGD4/R20 group

1 2 3 4 5 6

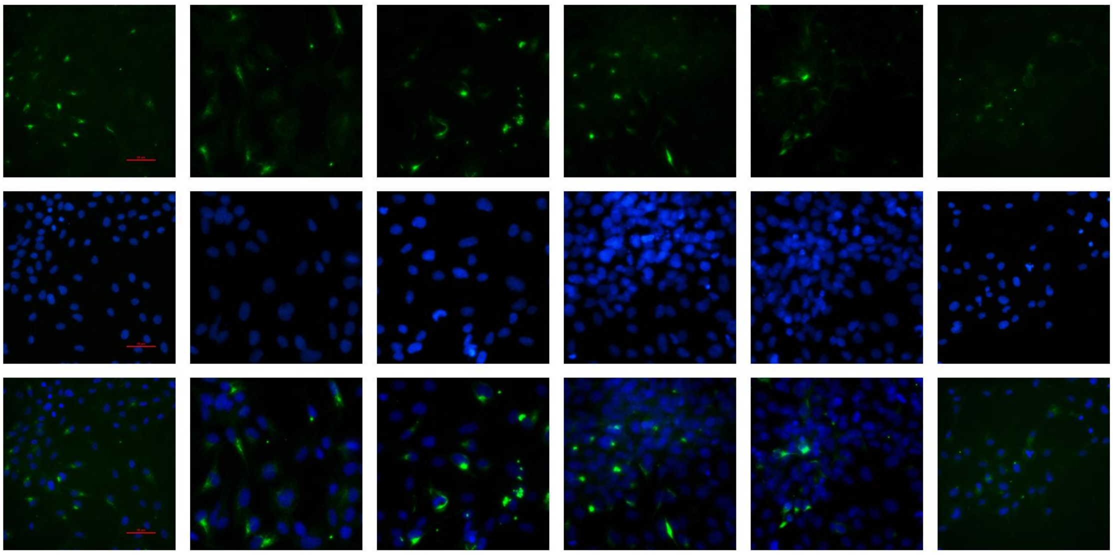

NC group of OGD4/R20

1 2 3 4 5 6

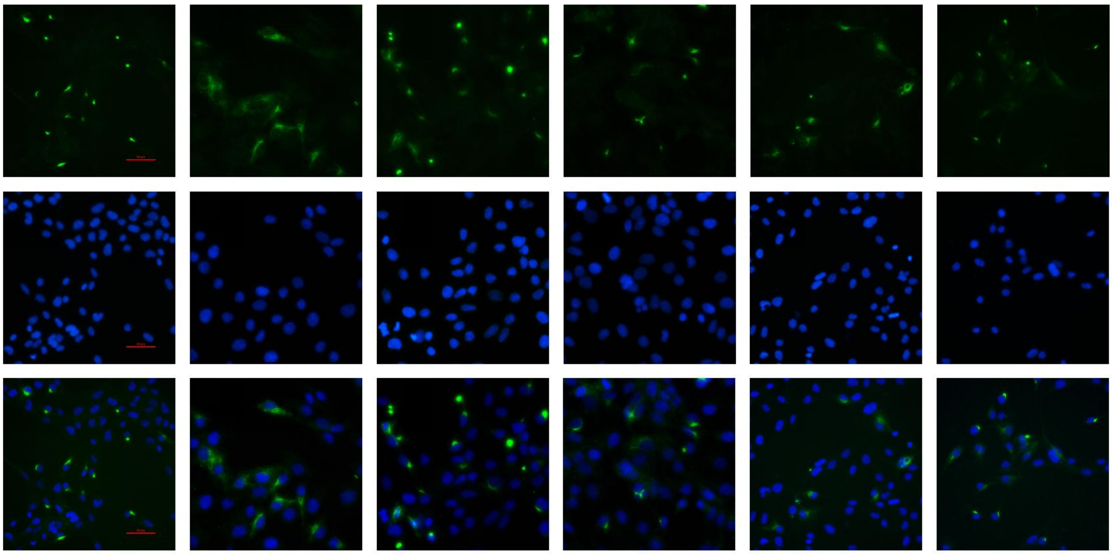

OE group of OGD4/R20

1 2 3 4 5 6

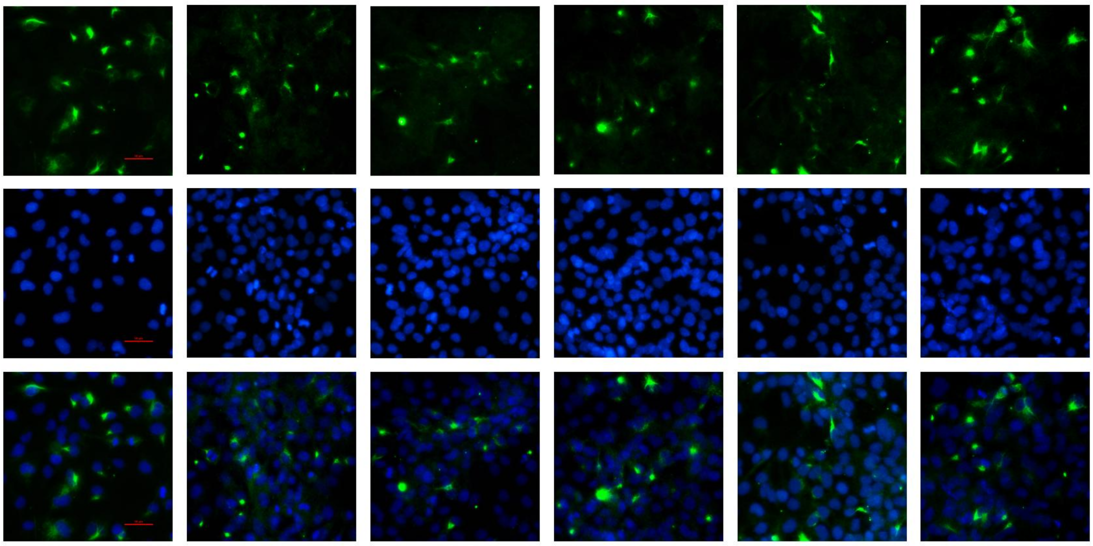

Fig.8C ZO1

Control group

1 2 3 4 5 6

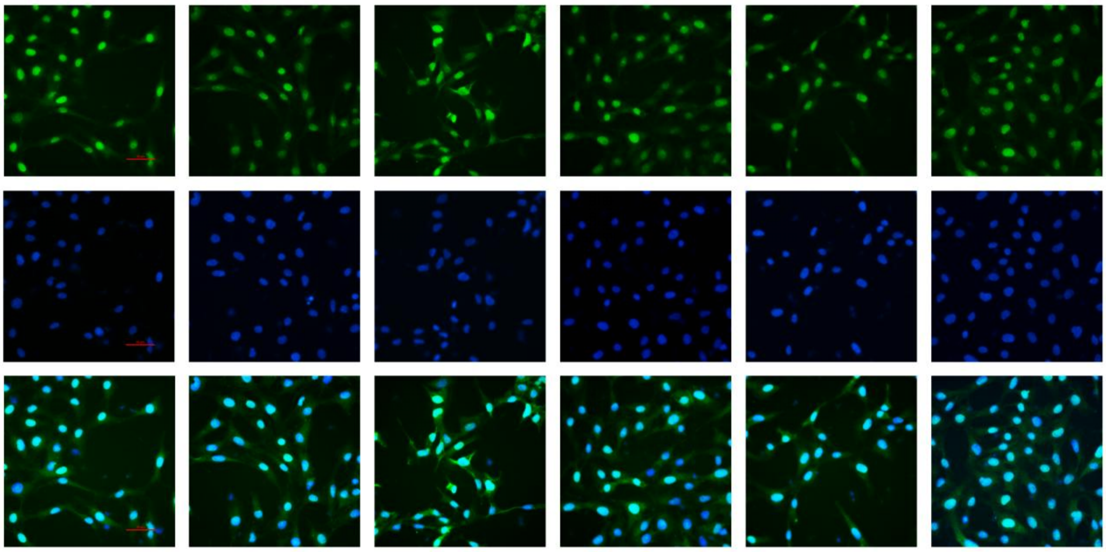

OGD4/R20 group

1 2 3 4 5 6

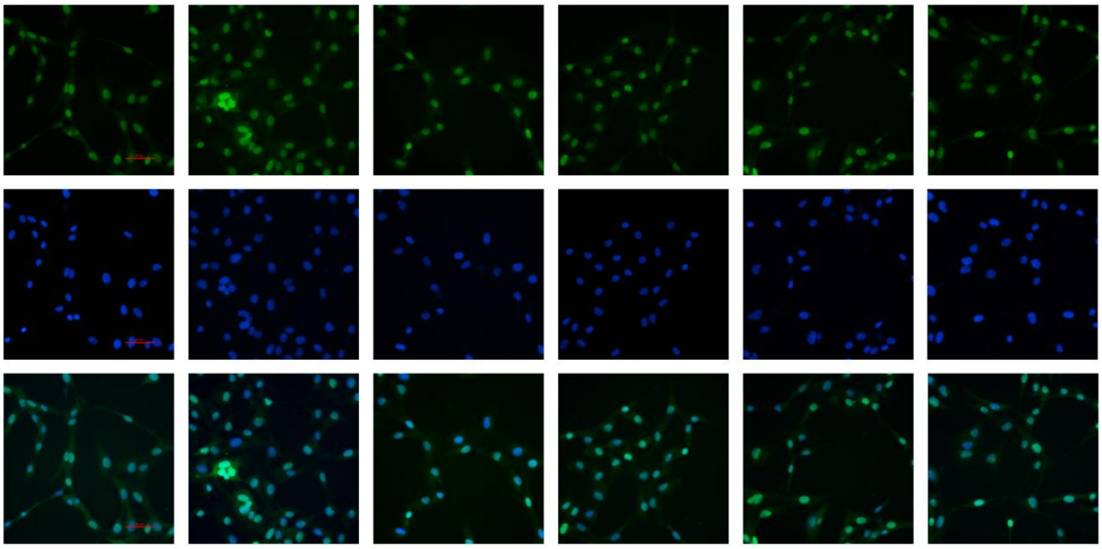

NC group of OGD4/R20

1 2 3 4 5 6

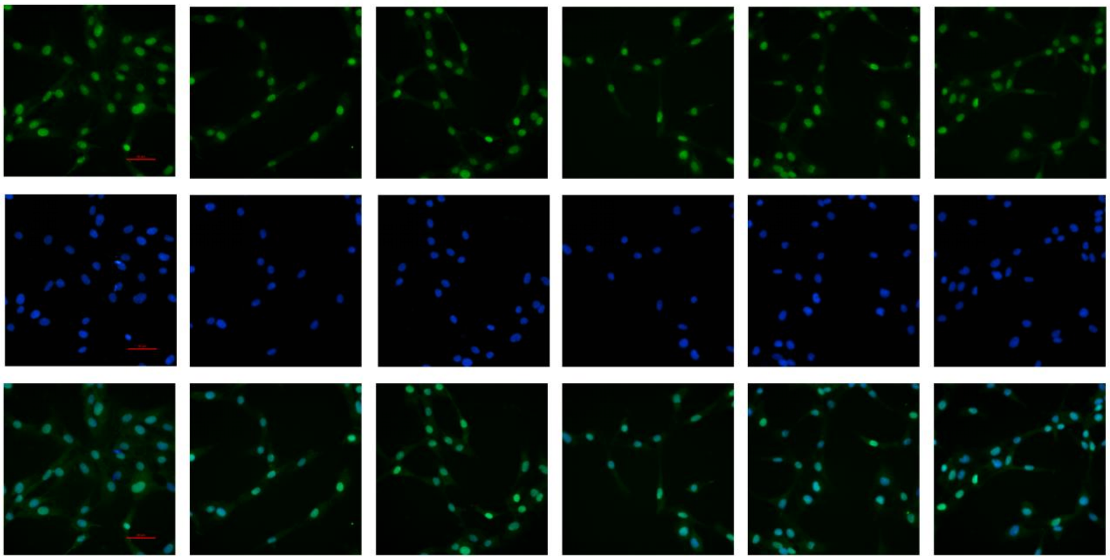

OE group of OGD4/R20

1 2 3 4 5 6

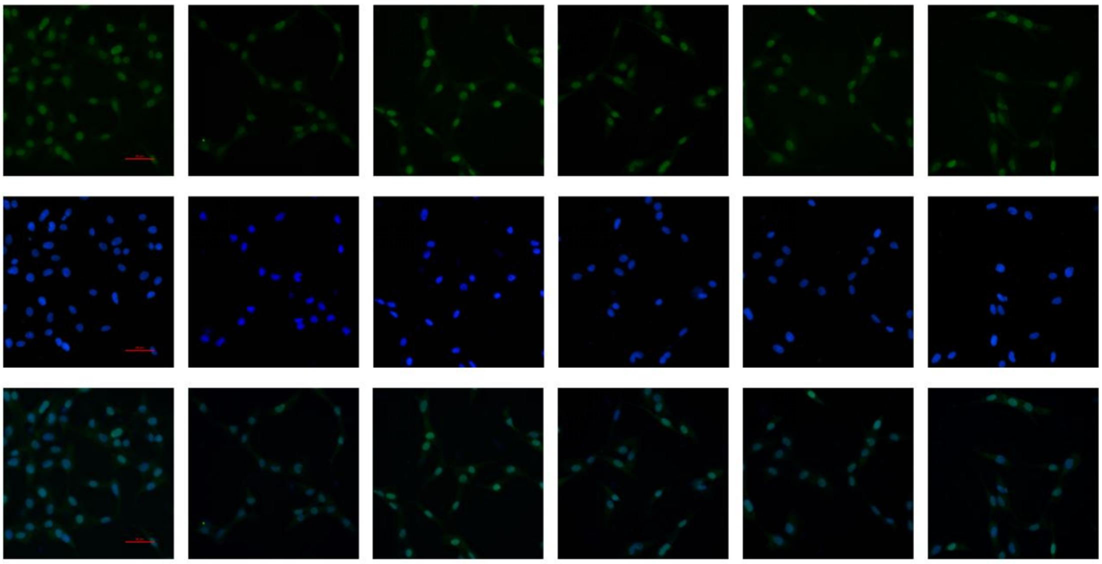

Fig.8D Occludin

Control group

1 2 3 4 5 6

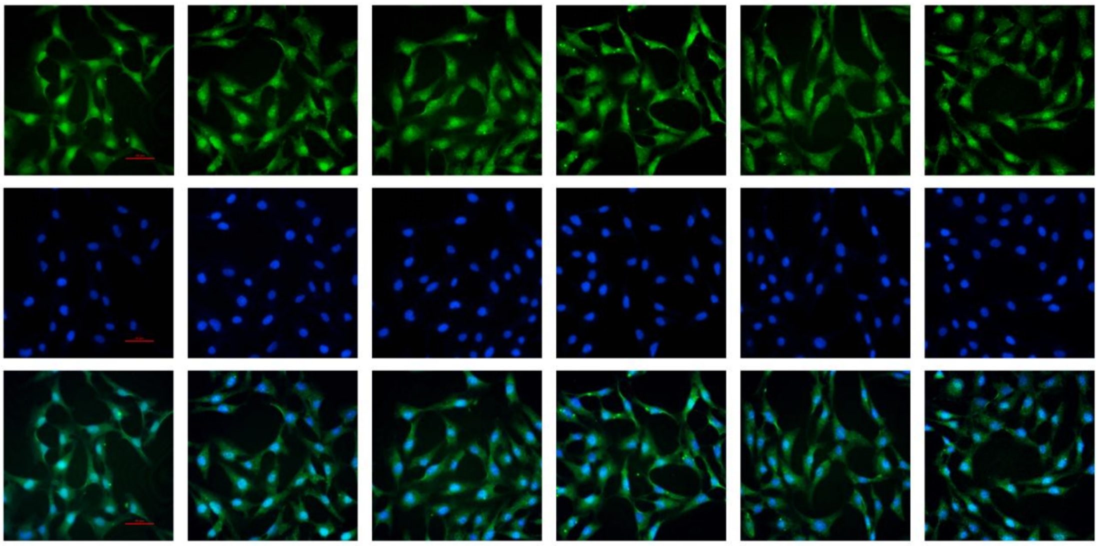

OGD4/R20 group

1 2 3 4 5 6

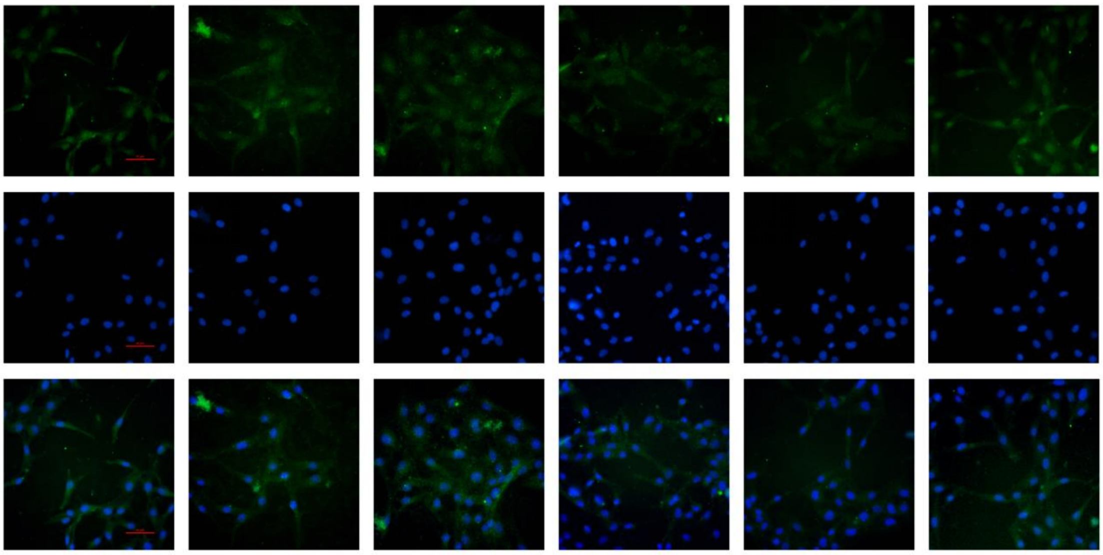

NC group of OGD4/R20

1 2 3 4 5 6

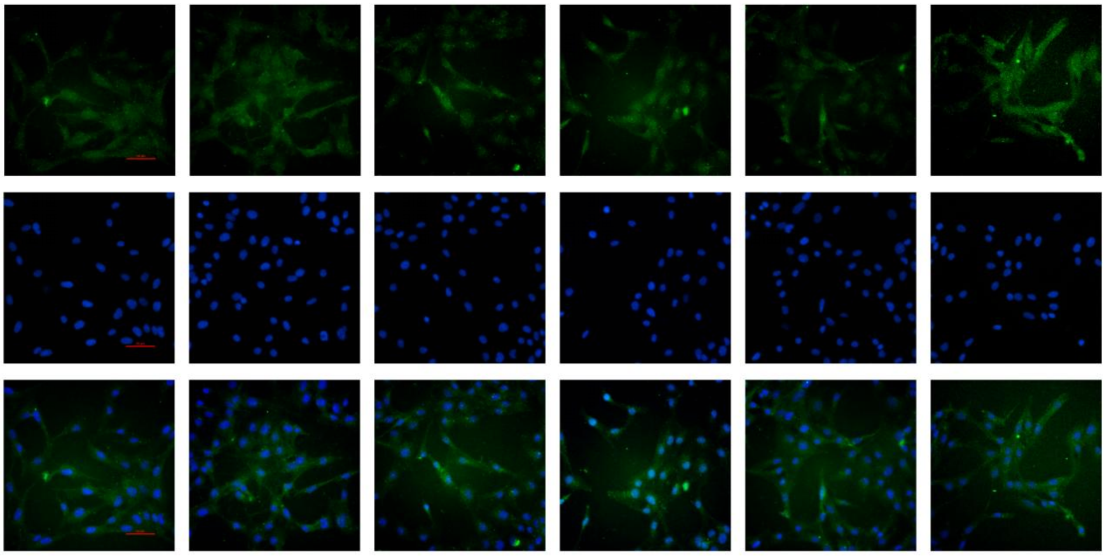

OE group of OGD4/R20

1 2 3 4 5 6

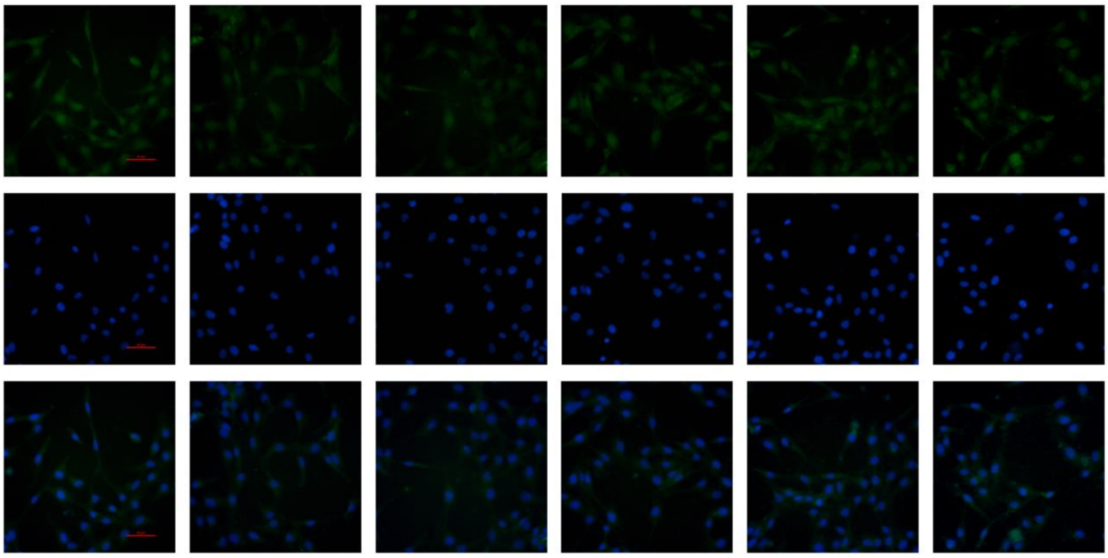

Fig.8E Claudin-5

Control group

1 2 3 4 5 6

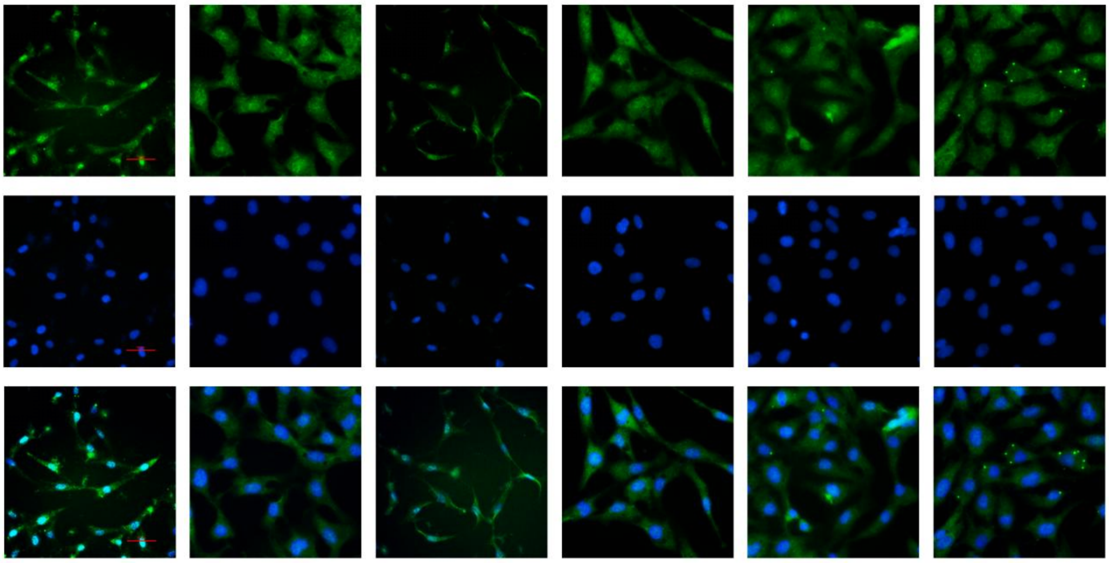

OGD4/R20 group

1 2 3 4 5 6

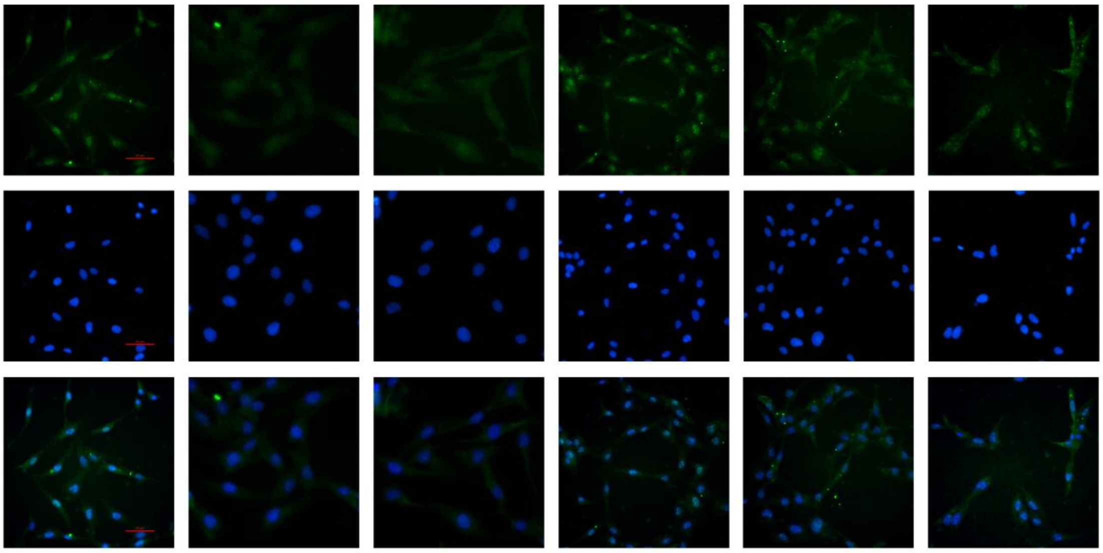

NC group of OGD4/R20

1 2 3 4 5 6

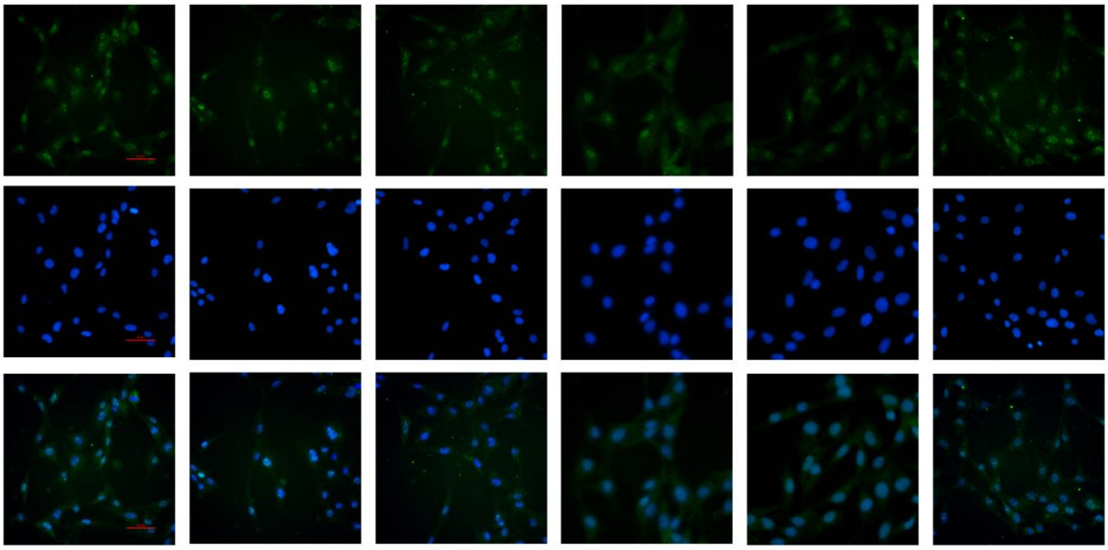

OE group of OGD4/R20

1 2 3 4 5 6

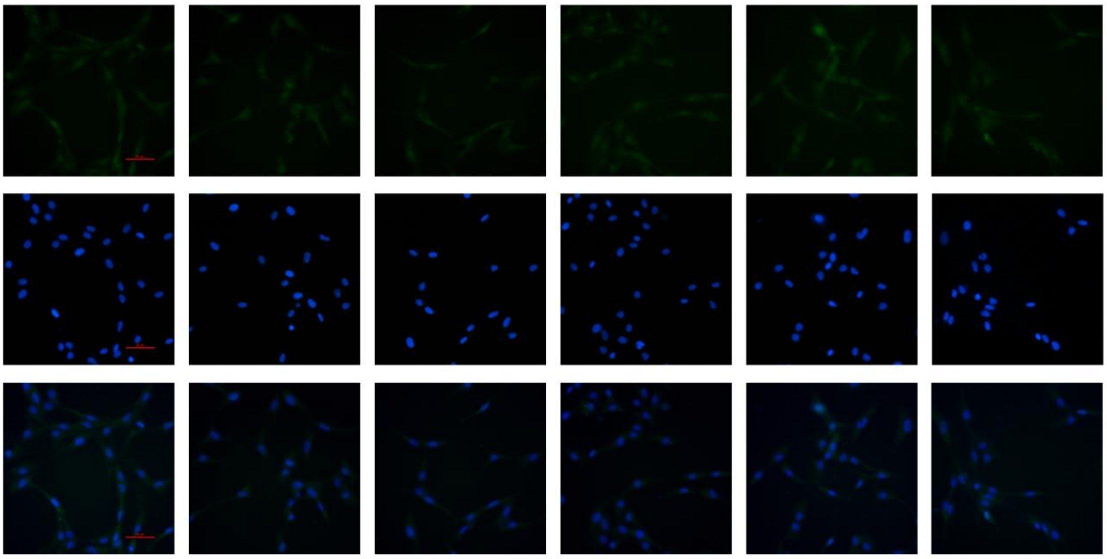

Supplement: Supplementary file 1 [file DataSheet2.zip › Raw data/raw picture.pdf]
