# Supplementary material for: The Novel lncRNA ENST00000530525 Affects ANO1, Contributing to Blood–Brain Barrier Injury in Cultured hCMEC/D3 Cells Under OGD/R Conditions
Source: Front Genet. 2022 Jun 8;13:873230. doi: 10.3389/fgene.2022.873230 (PMC9213740; doi:10.3389/fgene.2022.873230)
Supplement: Supplementary file 2 [file DataSheet1.docx]

Supplementary Table and Figure

**TABLE S1|** Primer sequences

| **Gene name** | **bidirectional primer sequence** | **annealing temperature (℃)** | **product length(bp)** |
| --- | --- | --- | --- |
| β-actin（H） | F:5' GTGGCCGAGGACTTTGATTG3'  R:5’ CCTGTAACAACGCATCTCATATT3’ | 60 | 73 |
| T294865 | F:5' TTGCCCTACACCTGCCTAT 3’  R:5’ CTCACTCCTCCCTACAAACACT 3’ | 60 | 105 |
| T058035 | F:5' CCTTGCCCGTAGTAGGTATTC 3’  R:5’ TGCCCAGCACAGTTATTCA 3’ | 60 | 150 |
| T150105 | F:5' TCGTTCCCAGACATCAAGAC 3’  R:5’ TGAAATCCAAACATGGTTAAGA 3’ | 60 | 95 |
| T131416 | F:5' TTGTTGGTTTATGGAACTGAGG 3’  R:5’ CTTGGCAAATGCTTTCACTC 3’ | 60 | 122 |
| ENST00000530525 | F:5' CTTCACTATCAGCAACCCCATT 3’  R:5’ GAACCTGCACTCTACGGTCAGC 3’ | 60 | 112 |
| ENST00000452599 | F:5' TCTGCCTCTTTGCATCCTACC 3’  R:5’ GCTCCTGCTGTCCAGTCACTC 3’ | 60 | 253 |
| ENST00000608826 | F:5' CCCTTTCTAAGAACTGACAGCC 3’  R:5’ AACAAAGAGCTGGAGGCAAT 3’ | 60 | 175 |
| T013651 | F:5' AGTCACGAAGGTCAAATAGCC 3’  R:5’ TGGCTTATCTTACCCACTCCT 3’ | 60 | 77 |
| T029143 | F:5' ATGTCATCCCGTCCTCCTCT 3’  R:5’ GGTCCTGAGATACCCCACAA 3’ | 60 | 118 |
| ENST00000527450 | F:5' AAGCTCCACTAACTGACCTGTG 3’  R:5’ GGATGACATGGTGATTACGG 3’ | 60 | 181 |
| ANO1 | F:5' AGGCCACACGGATCACAAAA 3’  R:5’ GGGAGAACCTGCACTCTACG 3’ | 60 | 40 |





**FIGURE S1|** LncRNA ENST00000530525 is Down-regulated in in stroke group compared with the control group by RT-qPCR. ***p<0.001. Control: n=20; stroke: n=40. Mann-Whitney U test.


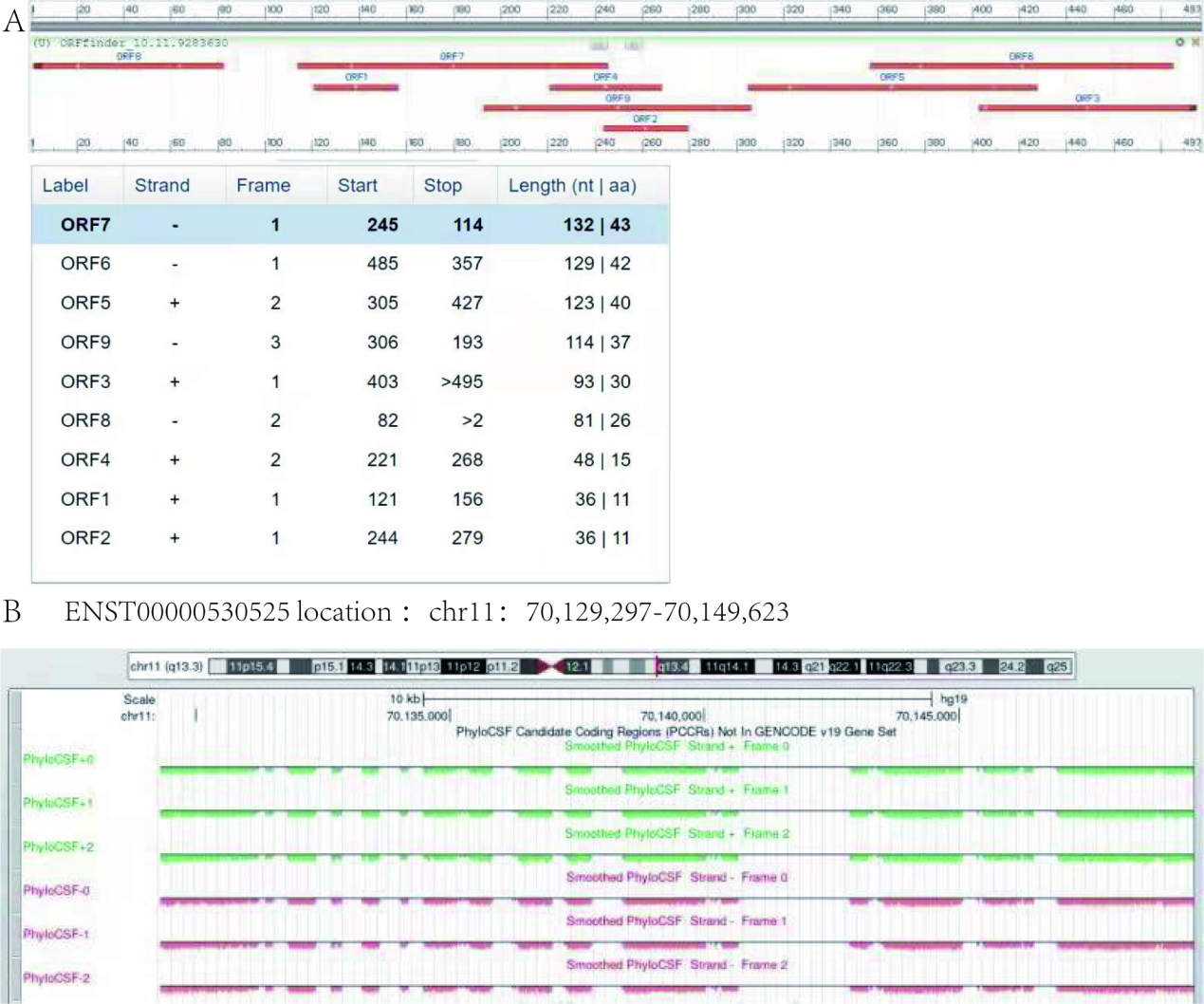


**FIGURE S2|** Non-coding nature of lncRNA ENST00000530525 was confirmed by coding-potential analysis.(A)Prediction of putative proteins encoded by lncRNA ENST00000530525 using ORF Finder. (B)The codon substitution frequency scores (CSF) of lncRNA ENST00000530525 were less than zero.


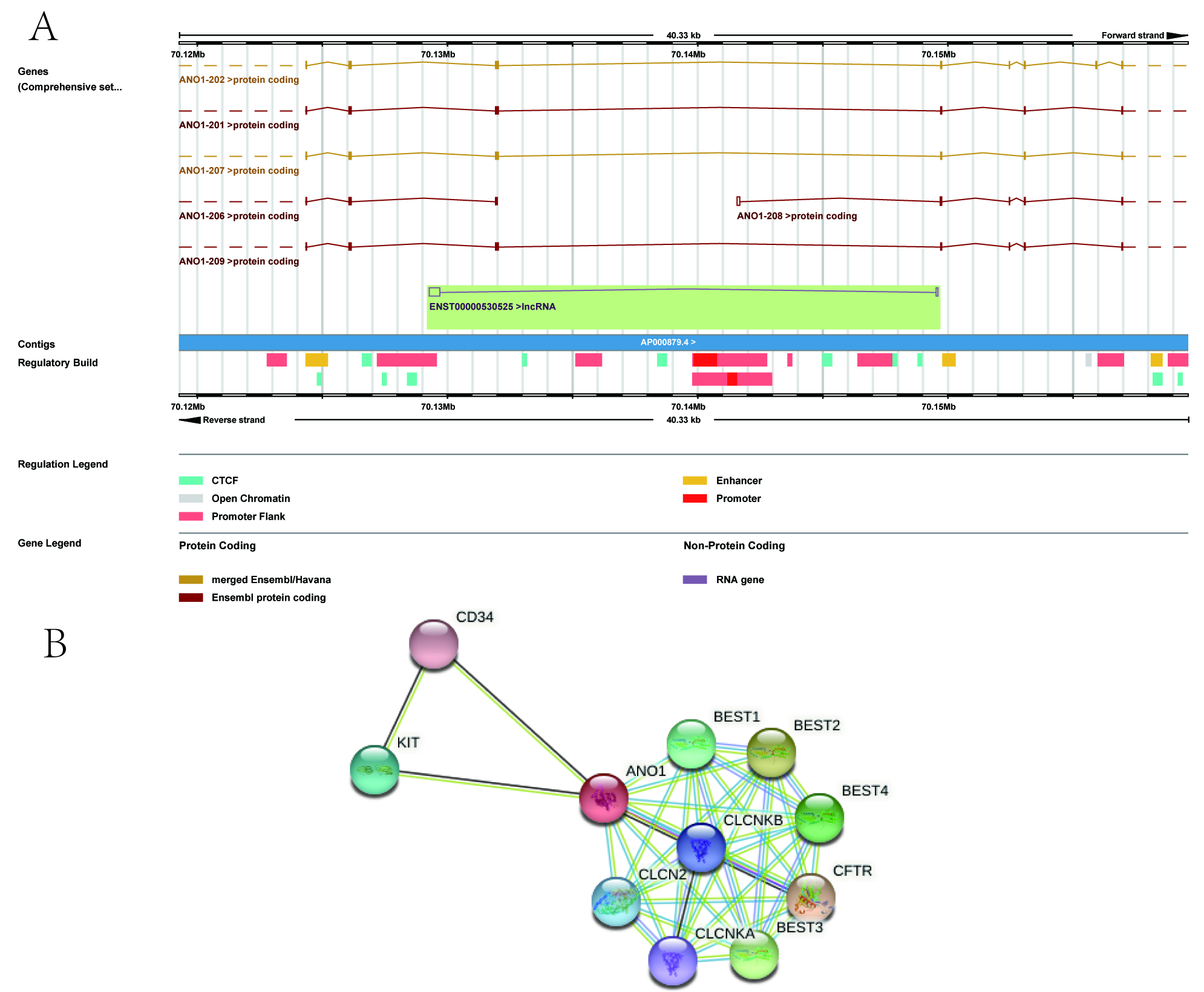


**FIGURE S3|** LncRNA ENST00000530525 is located intragenically with ANO1.(A) The schematic shows that lncRNA ENST00000530525 is within the anoctamin-1 (ANO1).(B)Online STRING analyses show the ANO1 protein-protein interaction network. ANO1 is closely related to CFTR.





**FIGURE S4|** Double luciferase assay was used to detect the regulation between lncRNA ENST00000530525 and target gene ANO1.Compared to Luc-ANO1-NC group, Luc-ANO1-mimic group has no significant difference when lncRNA ENST00000530525 was over-regulated(*P*<0.05 but the difference was less than 20%).*****P*<0.0001,####*P*<0.0001.
